# Cytotoxicity, anti-angiogenic, anti-tumor and molecular docking studies on phytochemicals isolated from *Polygonum hydropiper* L.

**DOI:** 10.1186/s12906-021-03411-1

**Published:** 2021-09-24

**Authors:** Mater H. Mahnashi, Yahya S. Alqahtani, Bandar A. Alyami, Ali O. Alqarni, Farhat Ullah, Abdul Wadood, Abdul Sadiq, Azam Shareef, Muhammad Ayaz

**Affiliations:** 1 Department of Pharmaceutical Chemistry, College of Pharmacy, Najran University, Najran, Kingdom of Saudi Arabia; 2 Department of Pharmacy, Faculty of Biological Sciences, University of Malakand, Chakdara, 18000 Dir (L) KP Pakistan; 3 Department of Biochemistry, Abdul Wali khan University, Mardan, KP 23200 Pakistan

**Keywords:** Breast cancer, HeLA cells, EGFR, HER2 receptors, VEGFR *Agrobacterium tumefaciens* NIH/3T3

## Abstract

**Background:**

According to the recent global cancer statistics, breast cancer is the leading cause of deaths among women with 2.3 million new cases globally. Likewise, cervical cancer is also among the leading causes of mortality among women. *Polygonum hydropiper* is traditionally known for its cytotoxic effects and several bioactive cytotoxic compounds were isolated from it. This study was aimed to isolate potential anticancer compounds from its most potent fractions and evaluate their anticancer potentials.

**Methods:**

Based on our earlier studies, active fractions including chloroform and ethyl acetate were subjected to column chromatography for isolation of compounds. Chemical structures of isolated compounds were confirmed via ^1^H NMR, ^13^C NMR, mass spectrometry. Purified compounds were tested for cytotoxicity against breast cancer cells (MCF-7), cervical cancer cells (HeLA) and NIH/3T3 fibroblasts cells cultures using MTT assy. Anti-angiogenic potentials of isolated compounds were evaluated via chorioallantoic membrane assay. Anti-tumor studies were done using *Agrobacterium tumefaciens* induced potato tumor assay. Furthermore, to understand the binding modes of Isolated compounds, molecular docking was performed against EGFR, HER2 and VEGFR using MOE as docking software.

**Results:**

Two bioactive compounds PH-1 (4-methyl-5-oxo-tetrahydrofuran-3-yl acetate) and PH-2 (methyl 4-hydroxy-3-methoxybenzoate) were purified from the active fractions. In cytotoxicity studies, PH-1 exhibited highest cytotoxicity against HeLA cells with 87.50% lethality at 1 mgmL^−1^ concentration and LD_50_ of 60 µgmL^−1^. Likewise, PH-2 showed 82.33% cytotoxicity against HeLA cells with LD_50_ of 160 µgmL^−1^. Similarly, PH-1 and PH-2 exhibited LD_50_ of 170 and 380 µgmL^−1^ respectively. Moreover, PH-1 and PH-2 were also very potent cytotoxic compounds against NIH/3T3 cells with 81.45 and 85.55% cytotoxicity at 1 mgL^−1^ concentration and LD_50_ of 140 and 58 µgL^−1^ respectively. Isolated compounds exhibited considerable anti-angiogenic potentials with IC_50_ of 340 and 500 µgL^−1^ respectively for PH-1 and PH-2. In anti-tumor assay, PH-1 and PH-2 exhibited 81.15 and 76.09% inhibitions with LD_50_ of 340 and 550 µgL^−1^ respectively. Both compounds selectively binds with EGFR and HER2 receptors with low binding energies. Both compounds exhibited stronger interactions with VEGFR through binding pocket residues Lys868, Val916 and Asp1046.

**Conclusions:**

Both compounds cause considerable cytotoxicity against cancer cells. The anti-angiogenic and anti-tumor results suggests additional tumor suppressive properties. Docking analysis suggests that these compound not only has the ability to bind to EGFR and HER2 but also equally binds to VEGFR and may act as potential anti-angiogenic agents.

**Supplementary Information:**

The online version contains supplementary material available at 10.1186/s12906-021-03411-1.

## Background

Breast cancer is the most prevalent cancers among women with high mortality rates [1]. The disease has high global burden with approximately 4.4 million cases worldwide and about 411,000 deaths annually which represent 15% of the total cancers deaths [2]. Its incidence varies greatly among different ethnic groups but generally age is a common influential factor. In north America, its incidence is 101.1 among 100,000 women and caused 40,110 deaths in 2004. However, its survival rate is increasing due to initial diagnose and proper therapy [3]. For instance, a data collected during 1989–2017 indicates that breast cancer related mortality is steadily decline by 40%. Yet another study indicates a slight annual increase of 0.3% in the new cases [4]. Likewise, cervical cancer is the second leading cause of deaths among women globally. It is expected to cause about 14,480 new cases with about 4290 deaths in 2021 (https://www.cancer.org/cancer/cervical-cancer/about/key-statistics.html). So both breast cancer and cervical cancers are among the major of cancer-induced deaths among female population. Currently available chemotherapeutics are associated with serve side effects [5, 6], so discovery and development of novel and safe drugs from natural products is necessary [7].

Various cancer cell lines are used worldwide to assess its pathobiology as well as efficacy of new investigational agents [8, 9]. Being a molecularly heterogeneous disorder, appropriate models are extremely necessary for prognosis of the diseases, underlying mechanism and drugs mechanism of action. Among the important benefits of using cell lines is their easy handling, cellular homogeneity and un-limited self replication [10]. Likewise, MCF-7 cells were established in 1973 at Michigan Cancer Foundation (MCF). And this cell lines is among the ideal models for breast cancer studies awing to their delicate sensitivity via expression of estrogen receptors (ER) [11]. Likewise, 3T3 cells were developed by S.A Aaronson and co-workers from mouse embryos [12].

Angiogenesis refers to the formation of new blood vessels and has got a significant role in the tumor proliferation [12]. Tumors with insufficient angiogenesis cannot achieve the logarithmic growth and remains dormant as tumor growth is particularly dependent on vascular growth which supplies required nutrients [13]. Subsequently, discovery of anti-angiogenic agents got considerable attention as tumor suppressive agents in cancer chemotherapy [14]. Several potential anti-angiogenic agents are in the process for novel drug discovery, yet no drug is currently approved for clinical use [15]. Chicken chorioallantoic membrane (CAM) is an ex-vivo tool to assess the effect of potential anti-angiogenic agents on the blood vessels formation [16]. Some medicinal plants especially traditional Chinese’s medicine are reported to have efficacy in ischemic diseases and cancer [17]. Likewise, potato tumor assay which is based on application *A. tumefaciens* containing tumor inducing gene is another in-vitro tool to analyze the preliminary anti-tumor potentials of agents [18].

*P. hydropiper* L. has about 50 genera and 1200 species which are important for their anticancer and diverse pharmacological properties [19–21]. Several species from *Polygonaceae* family are previously reported for cytotoxic potentials [22–25]. For instance, *Polygonum, Persicaria, Fallopia, Rumex* and *Oxyria* are reported to posses metabolites which hamper the proliferation of HeLA, MCF-7 cells [26]. Isolated compounds from *P. hydropiper* including warburganal and drimane type sesquiterpenoids like drimenol, polygodial, isodrimeninol, isopolygodial and confertifolin were reported for cytotoxicity [27]. In our previous studies we reported cytotoxic, anti-angiogenc and anti-tumor potentials of solvent extracts from the plant [12]. Subsequently we isolated several potential cytotoxic agents among which β-sitosterol and stigmasterol were reported for selective cytotoxicity [28]. In continuation of our previous work, we isolated two other potential compounds which were subjected to cytotoxicity against MCF-7, HeLA and NIH/3T3 as well as anti-angiogenic, anti-tumor and molecular docking with EGFR, HER2 and VEGFR for prediction of potential mode of binding.

## Materials and methods

### Plant collection and isolation of compounds

The selected medicinal plant, *P. hydropiper* which is traditionally famous for cytotoxic potentials is an annual wild herb which grows in marshy places at 22–25 °C. For the current study, *P. hydropiper* was collected in July 2013 from a marshy area in District Talash, Khyber Pakhtunkhwa (KP) after the permission of District Forest Administration Dir Lower KP. Subsequently, the plant was authenticated by botanical taxonomist Dr. Gul Rahim (Curator at the herbarium of University of Malakand). Dried plant sample was processed for preservation at the herbarium of University of Malakand for future reference with voucher no. H.UOM.BG.107. Whole research from plant collection to experimental work was carried out following Government of Pakistan, Ministry of Food, agriculture and co-operative department of plant protection legislations and provided guidelines (Supplementary file S2). Shade dried whole plant was subjected to fractionation as we reported in earlier studies [29–31]. Based on our previous evaluations of the crude extract and various fractions of *P. hydropiper* for different pharmacological activities [29–31], we set a preliminary target for the isolation of bioactive compounds. We aimed to isolate the bioactive compounds from chloroform and ethyl acetate fractions of the plant. Initially, we examined the fractions with pre-coated silica-based TLC plates to locate the possible phytochemicals using different eluent systems. We concluded that n-hexane and ethyl acetate solvent system was the best to locate the phytochemicals in the targeted fractions. Based on the amount of the crude fractions, a large gravity column was packed for the purifications/semi-purifications of bioactive compounds. We started elution with non-polar n-hexane and gradually increased the polarity with polar modifier ethyl acetate. We collected different groups of phytochemicals based on the co-elution of TLC R_f_ values. Furthermore, the dominant fractions from both ethyl acetate and chloroform fractions were combined separately. The two semi-purified fractions from chloroform and ethyl acetate were further subjected to relatively small silica packed columns. The small columns were eluted with the n-hexane and ethyl acetate solvent system carefully to purify the targeted compounds. At the end of both the pin-silica packed column, the two compounds (PH-1 and PH-2) were purified as visualized on post-column TLC analysis.

### Structure elucidation

Subsequent to compounds purification, any trace solvent was removed from the compounds using rotary evaporator. Firstly, ^1^H NMR was used to gain idea about the compounds structure and spectra were compared with already reported literature. Thereafter, ^13^C NMR analysis was carried out for analysis of carbon skeleton and the results were supplemented by mass spectrometry for confirmation of compounds structures.

### Ethical approval

The study protocol was approved by Departmental Research Ethics Committee (DREC), Department of Pharmacy, University of Malakand via reference no. DREC/20160502/01. All experiments were performed according to the rulings of the Institute of Laboratory Animal Resources, Commission on Life Sciences, National Research Council (1996) [31].

## Cell lines toxicity studies

### MCF-7 cell lines assay

Isolated compounds (32.25–1000 µgmL^−1^) were subjected to toxicity studies against breast cancer cells (MCF-7 ATCC® HTB-22™) following previously reported colorimetric MTT assay [32, 33]. Breast cancer cells were cultured in 96 wells microplate reader followed by overnight incubation at 37 °C using CO_2_ incubator. As stated in previous section, cells density was adjusted to 0.8 X 10^5^ cells mL^−1^ and were treated with 3–180 µM FLS for 24 h and then 48, 72 h sequentially followed by addition of 5 mg mL^−1^ MTT solution. Cellular mixture was again incubated and absorbance’s were recorded 570 nm using microplate reader (BioTek Instruments, Winooski, VT USA). Percent viability was elucidated from UV absorbance data and subsequently percent inhibition were determined using formula;

$${\text{Percent cells viability}} = {\text{Sample absorbance}}/{\text{Control absorbance X 1}}00$$

### HeLA cell lines assay

Both compounds (32.25–1000 µgmL^−1^) were tested against cervical cancer cells (HeLA ATCC® CCL2™) following previously reported colorimetric MTT assay [32, 34]. HeLA cells were cultured in sterilized MEME medium (Sigma-Aldrich) using 75 cm^2^ flasks and 5% FBS (Sigma-Aldrich), antibiotics including penicillin (100 IU mL^−1^) as well as streptomycin (100 µgmL^−1^) were used to prevent bacterial contamination the cultures. The cultures were incubated in CO_2_ incubator at 37 °C. After overnight incubation, fresh media supplemented with different concentrations of test samples were added to the cells cultures and incubated for 48 h. Thereafter, about 200 µl MTT solution was added to the culture and incubated for additional 4 h. After, 100 µl DMSO was transferred to each well and the concentration of reduced formazan in the cells cultures were assessed at 570 nm using micro plate reader (Spectra Max plus, Molecular Devices, CA, USA). Percent inhibition or cytotoxicity was calculated using formula;

$${1}00 - ({\text{Samples Abs}} - {\text{Control Abs}}/\left( {{\text{Positive control Abs}} - {\text{Negative control}}/{\text{Abs}}} \right){\text{ x 1}}00$$

### NIH/3T3 cell lines assay

Cytotoxicity of isolated compounds were also tested against Mouse embryonic fibroblast cells (ATCC® CRL-1658™) at the same concentrations following previously reported method [35]. Briefly, cells were cultured in DMEM medium added with 10% FBS. For the prevention of bacterial contamination, 50 IU mL^−1^ each of streptomycin and penicillin were added to the culture medium and incubated at 37 °C using CO_2_ incubator. NIH/3T3 cells were seeded in 96-well microplate reader with an adjusted cell density of 8.0 × 10^3^ cell/well (about 200 µl medium containing increasing concentrations of test samples) and incubated for 24 h. Cell culture free of test samples were used as negative control, whereas, cultures containing standard drug (doxorubicin) acted as positive control. Subsequently, 20 µl MTT solution having a concentration of 5 mg mL^−1^ prepared in PBS was added to each well and incubated for additional 4 h. Absorbance’s were recorded at 570 nm and from absorbance values cells viability was calculated and percent lethality was determined as follows;

$${\text{percent cells viability}} = {\text{ Treated groups absorbance}}/{\text{Control groups absorbance}}^{\prime}{\text{s}}\;{\text{X}}\;{1}00$$

### Anti-angiogenic assay

Samples were tested for their inhibitory effects on blood vessels formation using chorioallantoic membrane (CAM) assay [36]. Domestic chicken eggs were purchased from a local poultry trader in the vicinity if University of Malakand and were incubated at 37 °C for 4–7 days using humidified incubator to get fertilized. subsequent to incubation period, the formation of blood vessels were confirmed using flash light. A small hole was made at the narrow end of the egg and about one micro-liter albumin was withdrawn via sterilized syringe after which yolk sacs dropped away from shell membrane. On 8^th^ day of the experiment, a thermanox cover slip already loaded with required concentration of test samples as well as control drug were placed at the CAM surface and incubated again for 3 days. Later, acetone and methanol (1:1) was injected into CAM via 33 gauge needle so that CAM was alienated from eggs. The number of blood vessels were observed and counted in CAM for all groups under microscope.

Percent inhibition were calculated as = Blood vessels in CAM treated with control—Blood vessels in CAM treated with test samples / Blood vessels in CAM treated with control X 100.

Control: saline treated group

### Anti-tumor assay

Strain B6 of *A. tumefaciens* contains tumor inducing plasmid which when applied to potato discus causes formation of tumor outgrowths. The preliminary anti-tumor potentials of test compounds can be check using this approach. We tested isolated compounds using this anti-tumor model following already established protocol [37]. In brief, the microbe was cultured using SCDA (Soybean Casein Digest Agar) and incubated 25 °C overnight. The bacterial cultures were standardized to 1 X 10^8^ CFU. Solutions of both test compounds as well as control were prepared in DMSO. Negative control consists of 50 µL DMSO and 450 µL distilled water, Whereas, positive control contains the above solution with increasing concentrations of standard drug. For preparation of potato discs, red skinned fresh potatoes were obtained from local market. Potato discs (8 mm diameter and 2 mm height) were prepared using sterile cork borer. Prepared discs were washed with distilled water, surface sterilized via application of HgCl_2_ 1% solution for 4–5 min and again washed with distilled water. Discs were dried under sterile conditions for about 20 min and were placed equidistantly in pre-sterilized molten agar medium in plates using sterile forceps. Subsequently, surfaces of these discs were inoculated with bacteria and test compounds mixture. Plates were covered with parafilm and incubated in dark at 28 °C. subsequent to incubation for 15–20 days discs surfaces were stained with lugol’s solution and tumors were observed in all groups.

The antibacterial activity of our isolated compounds were also evaluated against *A. tumefaciens* using in-vitro disc diffusion assay [12, 38]. In brief, sterile discs impregnated with increasing concentrations of the compounds were equidistantly placed on nutrient agar plates inoculated and with *A. tumefaciens* and incubated overnight. Zone of inhibition around the discs were observed after 24 h of incubation at 37 °C using shaking incubator.

### Molecular docking studies against EGFR AND HER2 receptors

A molecular docking study was performed for both the compounds (PH-1 and PH-2) against epidermal growth factor receptor (EGFR) and human epidermal growth factor receptor (HER2) with PDB Code 4HJO and 3PP0 respectively. Next, the structural coordinate of the receptor was subjected to a molecular operating environment (MOE) software package for minimum energy conformation with the target receptor proteins for docking reason [39, 40]. Subsequently, 3D structures of our test compounds was generated with the MOE-builder module in MOE. Lastly, the optimized structures have been subjected to molecular docking using default molecular docking standard protocol in MOE. The top-ranking docked complex based on the protein–ligand interaction (PLI) profile was chosen for exploration of the binding mode. For ligand interaction and visualization refining protocol implemented in Pymol was used.

### Molecular docking against VEGFR

Molecular docking study of PH-1 and PH-2 against VEGFR (vascular endothelial growth factor receptor) was performed using by default docking protocol implemented in MOE 2016. The 3D structure of VEGFR (PDB ID 4AG8) was downloaded from protein data bank. The main purpose of the docking is to find out the binding behavior in terms of docking scores of PH-1 and PH-2 scaffolds against VEGFR and validate the role of VEGFR blockade in their anti-angiogenic and anticancer potentials.

### Statistical analysis

Experiments were performed in triplicates and data was shown as mean ± SEM. One way ANOVA followed by multiple comparison Dunnett’s test was applied for the statistical difference among all groups. *p* value < 0.05 were considered as statistically significant i.e. * *p*, 0.05,** *p* < 0.01 and *** *p* < 0.001 when compared with standard drug. Figures were generated in Graph Pad Prism software.

## Results and discussion

**Fig. 1:**
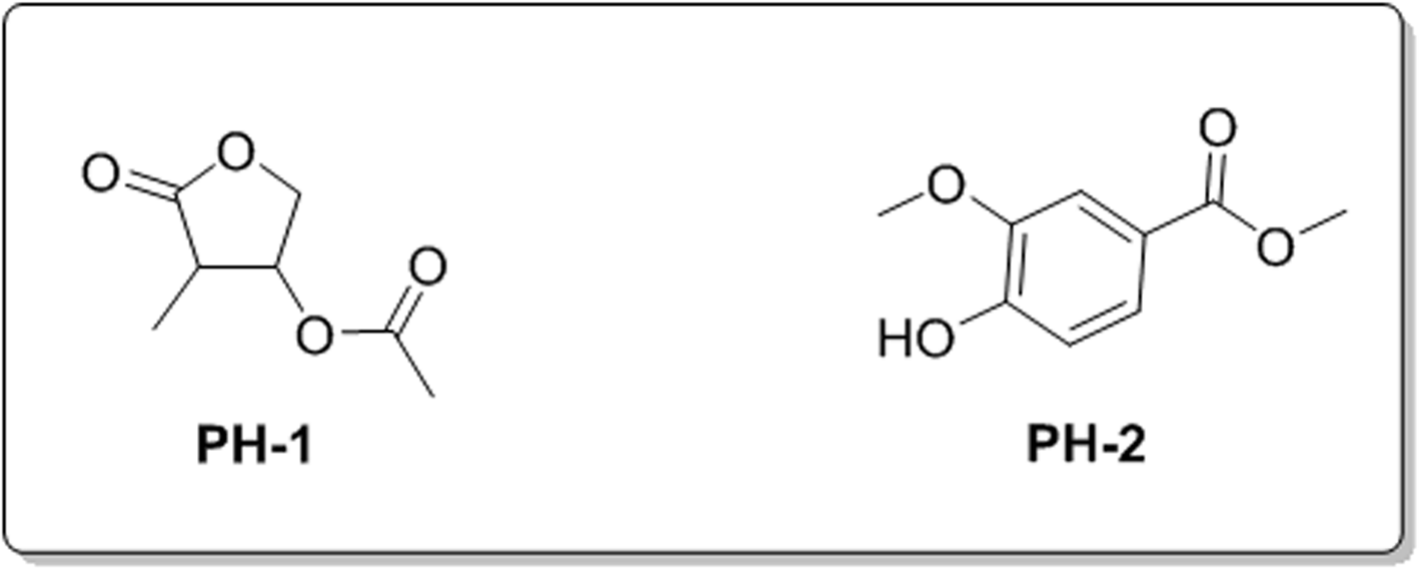
Chemical structures of the isolated compounds

In the current study, two bioactive compounds including PH-1 (4-methyl-5-oxotetrahydrofuran-3-yl acetate) and PH-2 (methyl 4-hydroxy-3-methoxybenzoate were isolated most active fraction of *P. hydropiper*,) as shown in Fig. 1. The compound PH-1 a substituted derivative of tetrahydrofuran which is semi-solid yellowish in color. The purified weight of the compound was 122 mg. On the TLC plate, PH-1 exhibited an Rf value of 0.28 with solvent system of *n*-hexane and ethyl acetate in a ratio of 80:20. In the ^1^H NMR spectrum, the methyl group directly attached to the tetrahydrofuran moiety gave a doublet of three protons at a chemical shift of 1.45. Similarly, the 2^nd^ methyl group of compound PH-1 gave a singlet at 2.11. Likewise, all the H-atoms of the tetrahydrofuran moiety were noted in the ^1^H NMR of the compound. The compound PH-2, a meta-para-substituted methyl ester derivative of benzoic acid was observed as Brown oil. The observed Rf value for this compound was 0.24 with eluting solvents of *n*-hexane and ethyl acetate (80:20). Obviously, the two methoxy groups appeared at 3.77 and 3.78 chemical shift values respectively. Similarly, the OH group and aromatic protons of the tri-substituted benzene ring were noted in the ^1^H NMR spectrum.

### Cell lines cytotoxicity

Cancer is broad term and refers to the uncontrolled proliferation of functionally abnormal cells which invades nearby tissues [41]. It is a leading cause of deaths globally and has a huge economic burden on health care system [42]. Several chemotherapeutic agents are clinically approved for the treatment of cancer but almost all are associated with high cost, severe adverse effects and toxicity issues [43]. Subsequently, natural products were explored for the discovery of more cost-effective and safe anti-cancer drugs. The discovery of lead drugs including vincristine and vinblastine from natural products is convincing evidence regarding the potentials of medicinal plants in anti-cancer drugs discovery [44, 45]. Keeping in view the role of natural products in anti-cancer drug discovery, the current project was designed to check the efficacy of isolated phytochemicals from an ethnomedicinally important plant *P. hydropiper* against various cancer cell lines. In the current study, PH-1 exhibited strong cytotoxic effects against breast cancer cells (MCF-7) causing 87.50% cytotoxicity at 1 mgmL^−1^ with LD_50_ of 60 µgmL^−1^ (Table 1). PH-2 also showed 82.33% cytotoxicity at 1 mgmL^−1^ concentration and LD _50_ of 160 µg mL^−1^. Likewise, PH-1 and PH-2 caused 77.25 and 71.90% cytotoxicity against HeLA cells respectively at the highest tested concentration of 1 mgmL^−1^. Their LD_50_ against HeLA cells were 170 and 380 µg mL^−1^ respectively. Both PH-1 and PH-2 displayed 81.45 and 85.55% cytotoxity respectively against NIH/3T3 cells at 1 mgmL^−1^ concentration. The LD_50_s against NIH/3T3 cells were 140 and 58 µg mL^−1^ respectively for both compounds. Standard drug doxorubicin showed 89.40, 92.00 and 88.53% cytotoxicity against NIH/3T3, HeLA and MCF-7 cell respectively. The LD_50_ against these cells were 15, 7 and 11 µgmL^−1^ respectively.

**Table 1:** Results of cytotoxicity studies on compounds PH-1 and PH-2

**Compound**	**Conc. µg mL**^**−1**^	**HeLA Cells**	**LD**_**50**_** µg mL**^**−1**^	**MCF-7 Cells**	**LD**_**50**_** µg mL**^**−1**^	**NIH/3T3 Cells**	**LD**_**50**_** µg mL**^**−1**^
PH-1	1000	77.25 ± 0.90^ns^	170	87.50 ± 0.86^ns^	60	81.45 ± 1.08^ns^	140
	500	63.45 ± 1.18**		74.58 ± 1.34^ns^		70.50 ± 0.57*	
	250	56.89 ± 0.92**		68.94 ± 1.03*		59.33 ± 0.728**	
	125	49.35 ± 0.93***		60.02 ± 1.15*		44.98 ± 0.43***	
	62.5	37.58 ± 0.79***		55.87 ± 0.64**		38.00 ± 1.15***	
	32.25	34.97 ± 0.72***		50.66 ± 1.37***		32.50 ± 0.57***	
PH-2	1000	71.97 ± 0.86*	380	82.33 ± 1.51^ns^	160	85.55 ± 0.79^ns^	58
	500	59.54 ± 1.18**		69.66 ± 0.79*		77.70 ± 0.50^ns^	
	250	42.38 ± 0.36***		57.71 ± 1.44**		70.50 ± 0.65*	
	125	37.60 ± 0.79***		54.38 ± 0.79**		61.45 ± 1.79**	
	62.5	33.80 ± 0.17***		49.59 ± 1.29***		55.00 ± 1.15***	
	32.25	29.50 ± 0.86***		30.91 ± 1.65***		41.90 ± 1.79***	

Data is presented in mean ± SEM after three experimental readings. Doxorubicin was used as positive control which revealed 89.40, 92.00 and 88.53% cytotoxicity against NIH/3T3, HeLa and MCF-7 cell respectively. The LD_50_ against these cells were 15, 7 and 11 µg mL^−1^ respectively. *P* value < 0.05 were considered as statistically significant i.e. * *p*, 0.05,** *p* < 0.01 and *** *p* < 0.001 when compared with standard drug, ns: results not significantly different in comparison to control group

Medicinal plants are playing a significant role in the drug discovery against various diseases including cancer [46, 47]. For instance, several anticancer drugs including vincristine, vinblastine, etoposide, topotecan, docetaxel, pacletaxel and irinotecan have been derived from natural sources and are effectively approved for clinical use. Among the families known for anti-cancer metabolites, *Polygonaceae* has got considerable importance and numerous species are reported for cytotoxic potentials [22–25]. Several species including *Polygonum, Persicaria, Fallopia, Rumex* and *Oxyria* are reported too posses metabolites which hamper the proliferation of HeLA, MCF-7 cells [26]. Several compounds isolated from *Rumex* species are reported to posses cytotoxic potentials [34, 48, 49]. Of particular importance is *P. hydropiper* which is extensively studied for the presence of cytotoxic compounds. Hong Xiao et al*.,* reported fourteen cytotoxic compounds from *P. hydropiper* [50]. Yet another group of researchers isolated warburganal and drimane type sesquiterpenoids including drimenol, polygodial, isodrimeninol, isopolygodial and confertifolin from the plant which possesses considerable cytotoxic potentials [27]. Plant is also reported for phytotoxic potentials [30]. We reported crude extracts and isolated compounds of *P. hydropiper* for MCF-7, HeLA, NIH/3T3 cytotoxicity, anti-angiogenic and anti-tumor potentials [12, 51]. The current work is an extension of our previous finding and the isolated compounds cause selective toxicities against HeLA, MCF-7, NIH/3T3 cells and inhibit blood vessels formation and tumor growth. The *in*-silico mode of cytotoxic action of the compounds in the cell lines is through inhibition of action EGFR and HER2 receptors as shown in molecular docking studies.

### Anti-angiogenic study

**Fig. 2:**
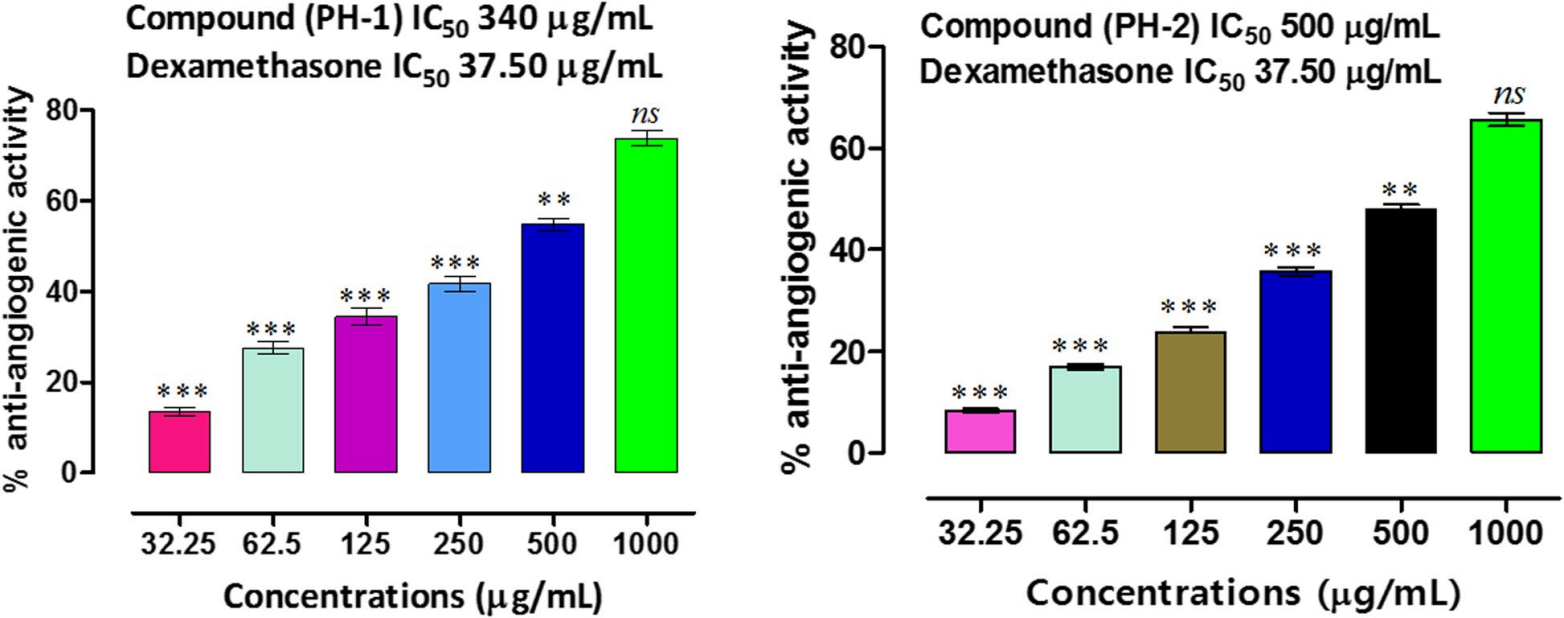
Anti-angiogenic potentials of isolated compounds using CAM assay. Results were expressed as percent inhibitions and Values represent mean ± SEM of three independent experimental readings. Dexamethasone was positive control whereas, distilled water was negative control. *p* value < 0.05 were considered as statistically significant i.e. * *p*, 0.05,** *p* < 0.01 and *** *p* < 0.001 when compared with standard drug

**Fig. 3:**
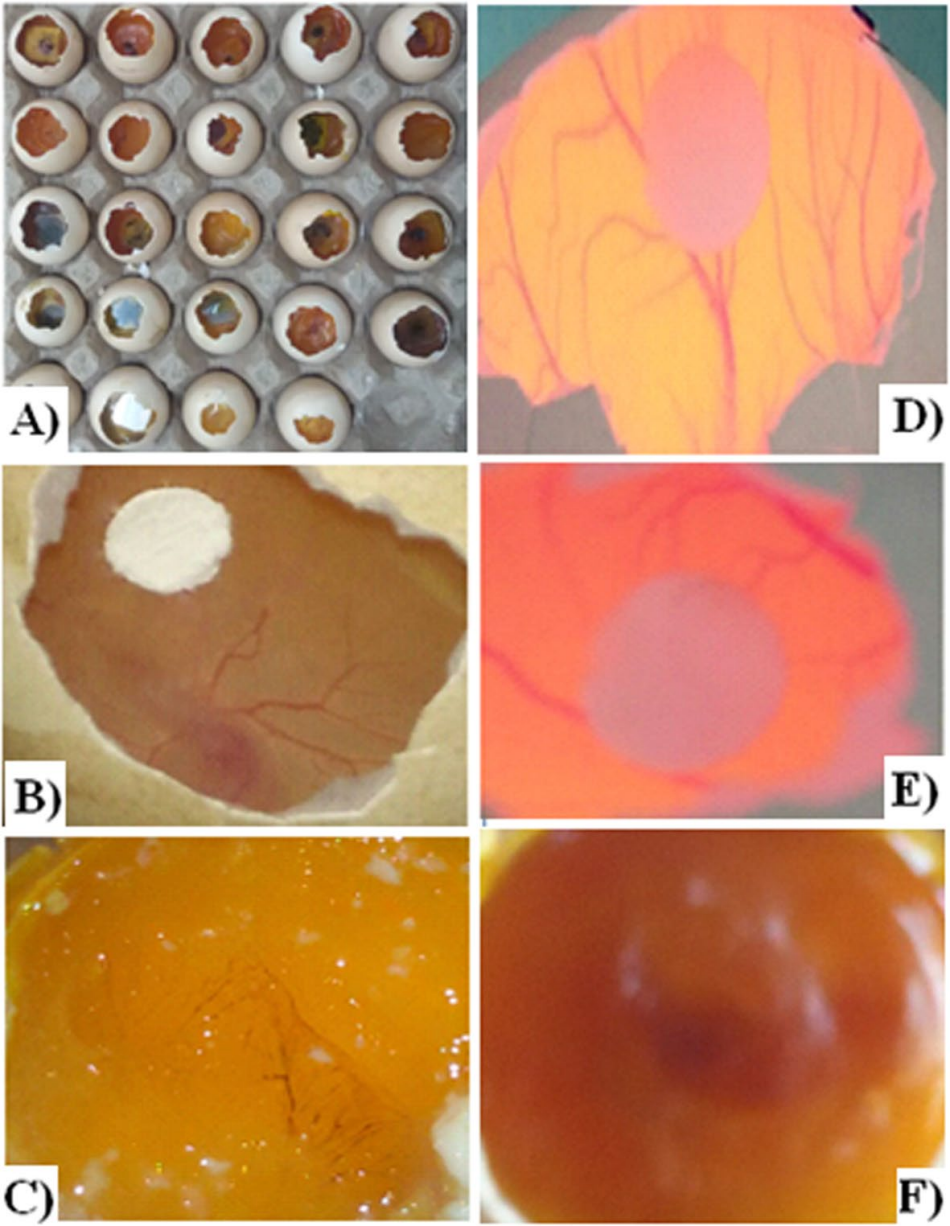
Images of Chorioallantoic membrane (CAM) assay. **A** Test samples incubated for assay. **B** Blood vessels inhibition in PH-1 treated group. **C** Blood vessels inhibition in PH-1 treated group on final day. **D** Blood vessels in negative control group. **E** Blood vessels inhibition in PH-2 treated group. **F** Blood vessels inhibition in PH-2 treated group on final day of data collection

Angiogenesis is the formation of new blood vessels which are required for the rapid growth of the tissues and to meet the nutritional needs of rapidly growing cells. Anti-angiogenic agents have got significant attention as these agents suppress blood vessels formation which are required for rapid growth of tumors. Thus tumors after anti-angiogenic therapy remains dormant. Whereas, drugs which stimulate angiogenic process have applications in ischemic heart diseases. Subsequently, the use of anti-angiogenic agents is among the vital strategies of oncologists. Chorioallantoic membrane (CAM) is chicken embryo based assay which is an important model to study the anti-angiogenic effects of test samples. In this method, angiogenic response is stimulated in 72–96 h and blood vessels are formed radiating towards the center of the implant. Test drugs are applied at this stage which inhibit the blood vessels formation and proliferation. In CAM anti-angiogenic assay, PH-1 exhibited concentration dependent inhibitions of blood vessels formation. PH-1 caused 13.47, 27.62, 34.50, 41.66, 54.75 and 73.83% inhibitions at tested concentrations of 32.25, 62.5, 125, 250, 500 and 1000 µgmL^−1^ respectively (Figs. 2 and 3). The IC_50_ value for PH-1 was 340 µgmL^−1^, whereas, positive control drug dexamethasone showed IC_50_ of 37.50 µgmL^−1^. Likewise, PH-2 showed 8.35, 16.98, 23.81, 35.75, 47.89 and 65.64% inhibitions at the same tested concentrations respectively with IC_50_ of 500 µgmL^−1^. Several natural anti-agiogenic bioactives are previously reported from medicinal plants which have the benefit of low toxicities and better efficacy in comparison to synthetic chemotherapeutic agents [17, 52]. Results of the current study provides additional mechanism of potential anticancer applications of the isolated compounds.

### Tumor inhibition assay

**Fig. 4:**
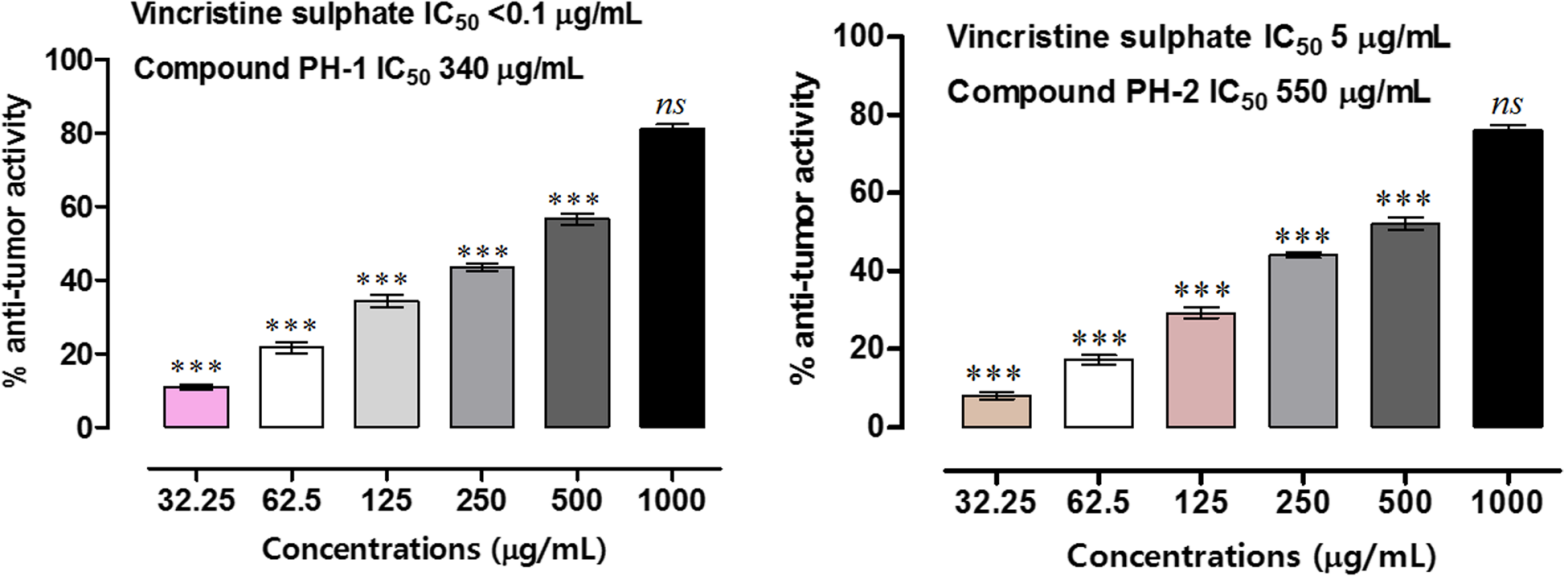
Results of the anti-tumor assay. Values represent mean ± SEM of three independent experimental readings. Vincristine sulphate was used as positive control whereas, DMSO was used as negative control. *p* value < 0.05 were considered as statistically significant i.e. * *p*, 0.05,** *p* < 0.01 and *** *p* < 0.001 when compared with standard drug

**Fig. 5:**
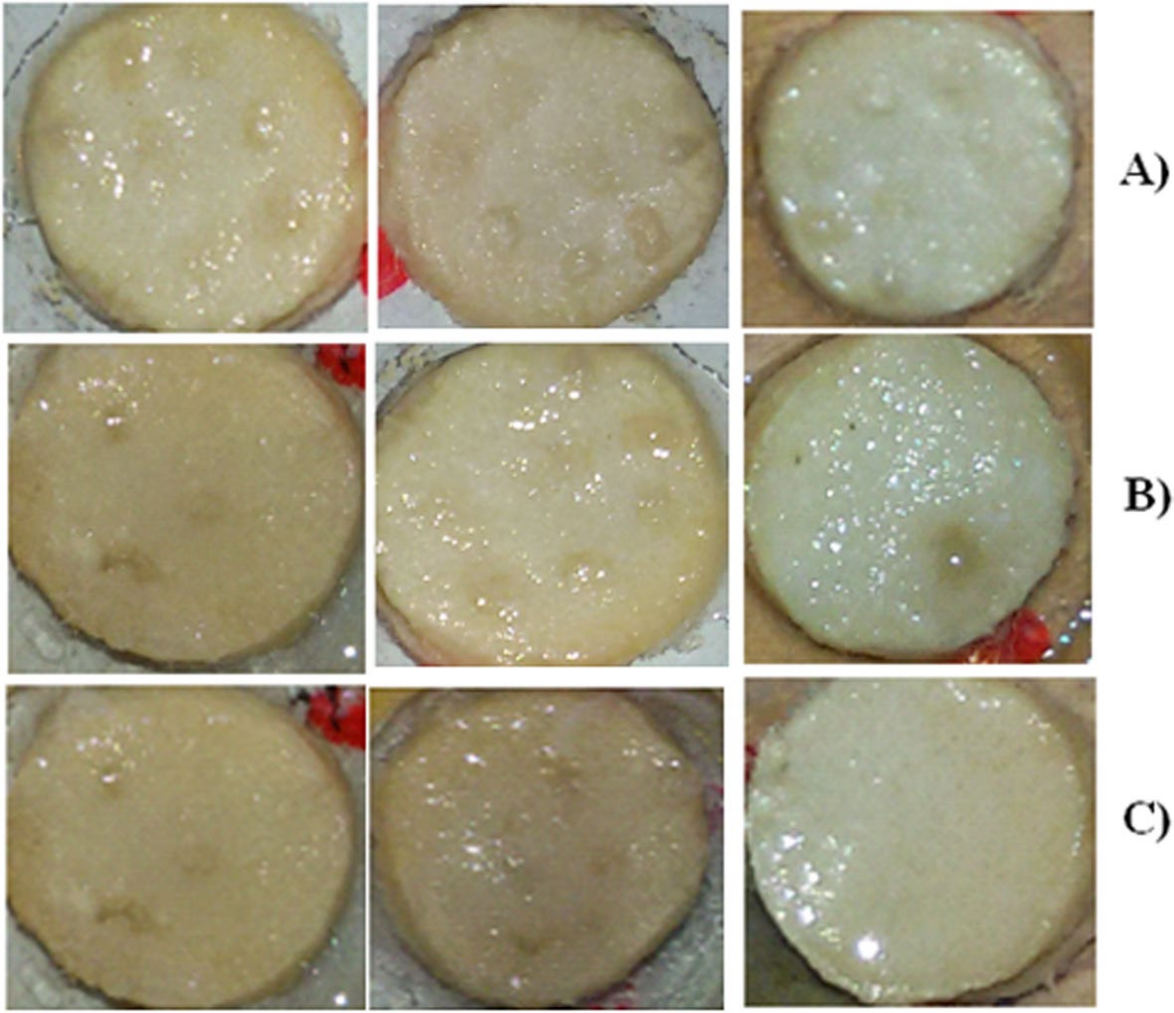
Representative images of *Agrobacterium tumefaciens* induced potato tumor assay. **A** Tumor formation after application of *A. tumefaciens* solution and incubation. **B** Gradual decline in the number of potato tumors. **C** Tumors on data collection day

Potato disc anti-tumor assay is a rapid and reliable preliminary tool to assess the efficacy of test samples for potential anti-cancer applications. This assay is an anti-mitotic activity and represent a variety of anti-tumor effects [37]. The tumor formation is a neoplastic diseases of plants induced by *A. tumefaciens*. The bacteria contains tumor inducing plasmids thus carrying genetic information’s (T-DNA) which subsequent to infections in plants cause convert normal cells to independent tumors [18, 53]. The plasmids cause the plant cells to multiply rapidly without going through apoptosis and thus leads to the formation of large size tumors. These tumors have high similarity with animals and human cancers with respect to histology, nucleic acid contents [54]. Thus an additional support to the current cytotoxic results were provided by appraising the anti-tumor potentials of isolated compounds. In our study, both compounds displayed considerable inhibitions of potato tumors. PH-1 showed 11.07, 21.69, 34.43, 43.51, 56.76 and 81.15% inhibitions at 32.25, 62.5, 125, 250, 500 and 1000 µgmL^−1^ respectively as shown in (Figs. 4 and 5). Likewise, PH-2 showed 8.11, 17.27, 29.33, 43.48, 52.07 and 76.09% inhibitions at the same concentrations respectively. IC_50_s for PH-1, PH-2 and positive control were 340, 550 and 5 µgmL^−1^ respectively. Medicinal plants are previously reported to posses considerable anti-tumor potentials using the same paradigm [55, 56].

In our initial antibacterial evaluations using disc diffusion assay, which was aimed to check the antibacterial activity of test compounds against tumor inducing bacterial strain revealed absolutely no antibacterial activity at the tested concentrations. These antibacterial evaluations are required to check as if the tested samples inhibit bacteria responsible for tumor induction and thus effect their inherent ability to induce tumors. Compounds having inhibitory activity against *A. tumefaciens* are not good candidates for checking their anti-tumor efficacy using this model [12].

### Molecular docking studies against EGFR and HER2 receptors

To better explore the connection of our cytotocicty results and binding affinities of our samples with the target proteins, a computational approach i.e. molecular docking analysis of the titled 4-methyl-5-oxo-tetrahydrofuran-3-yl acetate and methyl 4-hydroxy-3-methoxybenzoate against EGFR and HER2 was carried out via default docking protocol of MOE 2016. Keeping the importance of these proteins i.e. EGFR, HER2 are absolutely important targets to discover new cytotoxic agents. Additionally, EGFR is a key cell-surface receptor for epidermal growth factor family and animated by interacting of its proper ligands [57]. It has been also found with mandatory role in the growth of ductal system of the mammary glands [58]. Furthermore, the consequences of over expression of EGFR is large number of cancers including squamous-cell carcinoma of the lung, anal cancers [59], glioblastoma and epithelial tumors of the neck and head [60]. Similarly HER2 is of equal importance and has been an important member of human epidermal growth factor receptor (HER/EGFR/ERBB) family, but still it’s over expression causes a number of dangerous and fatal types of cancers such as ovarian [61], breast, stomach, adeno-carcinoma of lungs [61] and uterine cancer [62, 63]. On the basis of such importance of these proteins we have chosen them as target receptors for our docking protocol. Here our objective was to find out the binding behavior in terms of docking scores of synthesized 4-methyl-5-oxo-tetrahydrofuran-3-yl acetate and methyl 4-hydroxy-3-methoxybenzoate scaffolds against target receptors (EGFR/HER2) and subsequently compare our findings with well-known inhibitors of these receptors like gefitinib (EFGR), lapatanib (EGFR), afatinib (HER2) and canertinib (HER2). Docking results of our samples and their comparison with previously reported standard agents is summarized in Tables 2 and 3 respectively.

**Table 2:** Interaction detail of EGFR with 4-methyl-5-oxo-tetrahydrofuran-3-yl acetate and methyl 4-hydroxy-3-methoxybenzoate

**Compound**	**Docking Score**	**Interactions details**							
		Ligand		Receptor (EGFR)			Interaction	Distance	E(kcal/mol)
PH-1	-5.4888	C	5	OG1	THR	766	H-donor	3.18	-0.6
		O	6	NZ	LYS	721	H-acceptor	3.19	-9.3
		O	10	OG1	THR	830	H-acceptor	2.98	-2.0
		O	10	N	ASP	831	H-acceptor	2.94	-1.3
PH-2	-4.9394	O	7	OG1	THR	766	H-donor	2.99	-2.0
Geftimib	-7.2019	N	11	CA	ASP	831	H-acceptor	3.64	-0.6
Lapatinib	-7.5156	C	56	OG1	THR	830	H-donor	3.38	-0.5
		6-ring		CB	LEU	694	Pi-H	3.99	-0.7

**Table 3:** Interactions details of HER2 with 4-methyl-5-oxo-tetrahydrofuran-3-yl acetate and methyl 4-hydroxy-3-methoxybenzoate

Compound	**Docking Score**	**Interactions details**							
		Ligand		Receptor (HER2)			Interact	Distance	E(kcal/mol)
PH-1	-4.9760	O	6	N	MET	801	H-acceptor	3.08	-2.8
PH-2	-5.5790	O	7	OD2	ASP	863	H-donor	3.03	-3.0
		6-ring		CG2	VAL	734	Pi-H	3.84	-1.1
Canertinib	-7.3353	6-ring		CG2	VAL	734	Pi-H	4.07	-0.5
Affitinibe					ASP	763			

**Fig. 6:**
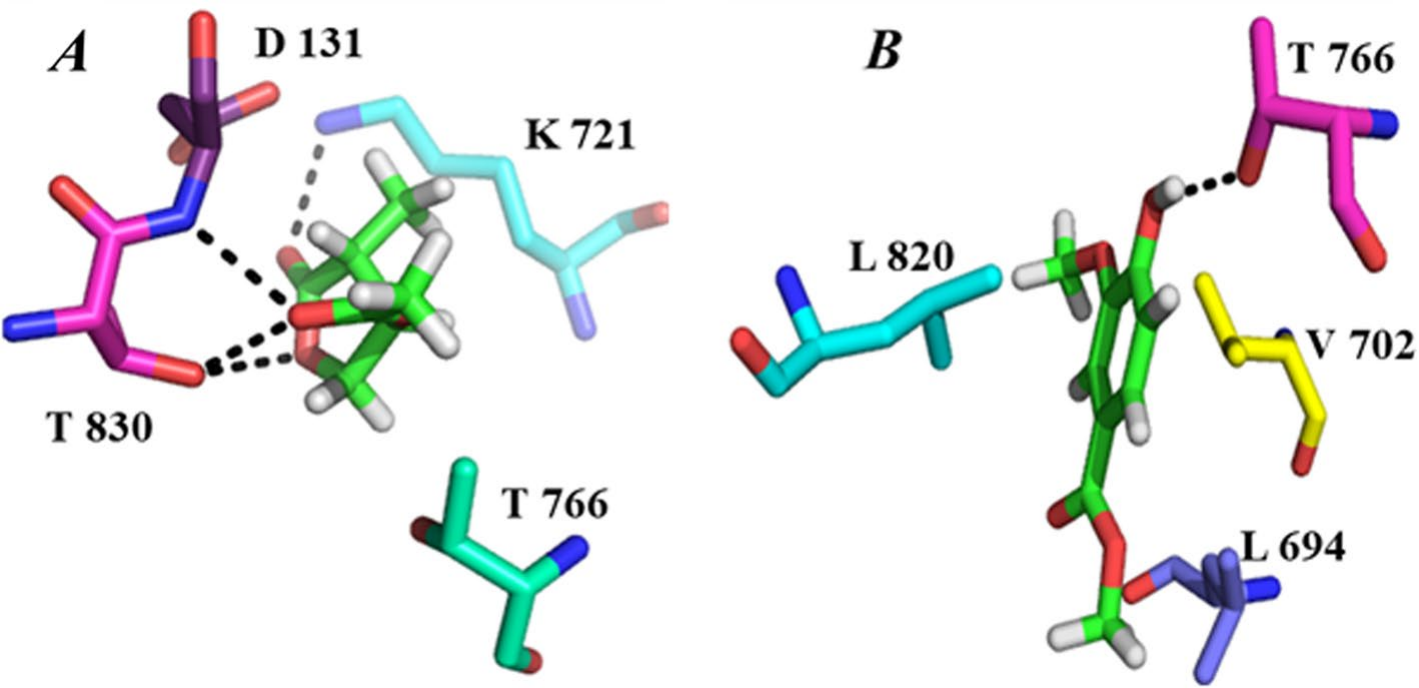
**A**, **B** Binding interactions of compounds with EGFR. **A** Shows binding interaction of PH-1, whereas **B** shows binding interaction of PH-2, the green color represent the ligand

As interaction detail given in the Table 2 indicated that both the tested compounds have better interaction with EGFR as compare to gefitinib and lapatanib especially 4-methyl-5-oxo-tetrahydrofuran-3-yl acetate, it has four strong hydrogen bonding with binding pocket residues i.e. THR 766, LYS 721, THR 830 and ASP 831 along with other hydrophobic interactions as shown in Fig. 6A. The compound has two highly electron withdrawing carbonyl oxygen i.e. Oxygen 6 and Oxygen 10. Oxygen 6 develop hydrogen bonding with NZ of Lys 721 and oxygen 10 formed two strong intermolecular interactions with OG1 of THR 830 and with nitrogen of Asp 831 giving extra stability to complex. The carbon 5 of compound also form a non-polar interaction with THR 766 making this complex thermodynamically more stable and hence may have the ability to be an inhibitor for over expression of EGFR. Similarly the 2nd compound i.e. methyl 4-hydroxy-3-methoxybenzoate although do not have much interactions and better docking score but still comparable with reference compounds. Methyl 4-hydroxy-3-methoxybenzoate has strong hydrogen bonding with THR 766 of active site along with hydrophobic interactions with binding pocket residues like VAL 702, LEU 694 and LEU 820 as shown in Fig. 6B. The hydroxyl group of the compound makes the compound to highly interact with receptor. The hydroxyl group of the compound interacts with active site residue THR 766 moving the system toward stability.

**Fig. 7:**
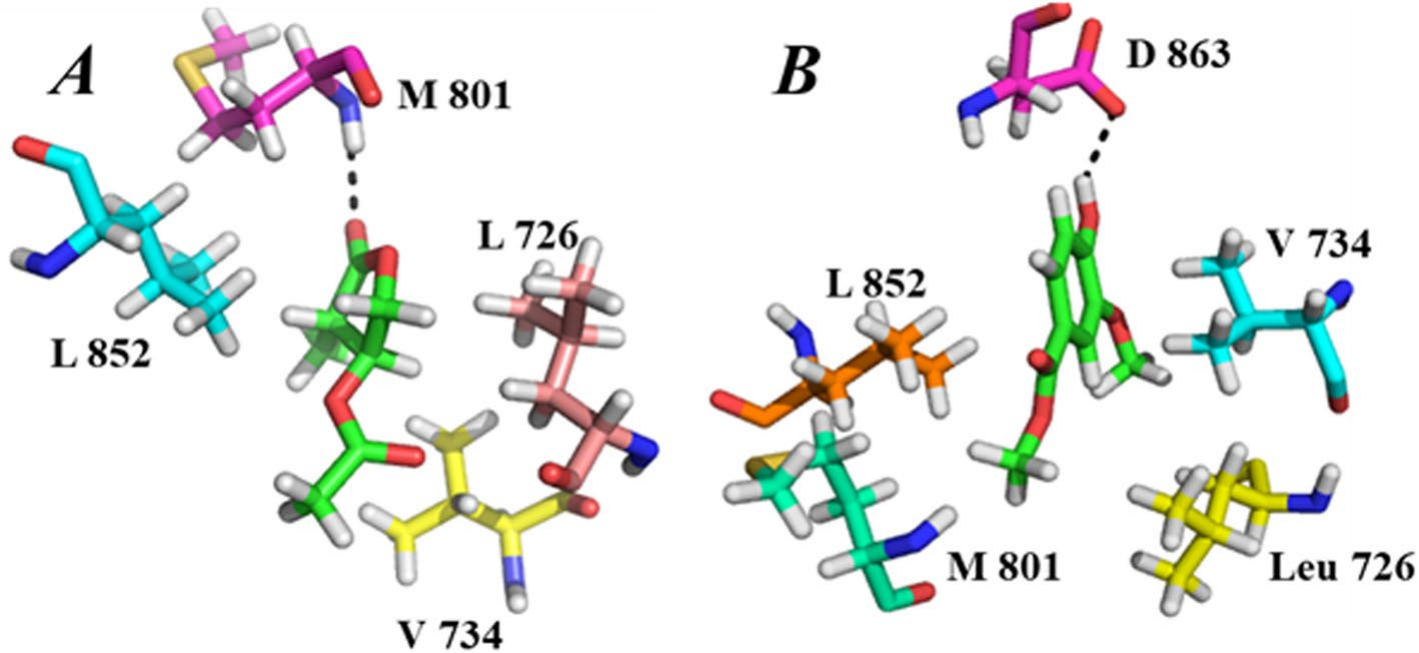
**A**, **B** Binding interactions of compounds with HER2. **A** Shows binding interaction of 4-methyl-5-oxo-tetrahydrofuran-3-yl acetate whereas **B** shows binding interaction of methyl 4-hydroxy-3-methoxybenzoate, the green color represent the ligand

Likewise EGFR these compound also give satisfactory results with HER2 target and may be better inhibitor than its reference compound afatinib and canertinib, interestingly with HER2 the 2^nd^ compound i.e. methyl 4-hydroxy-3-methoxybenzoate have better docking score, interaction detail like binding energy and distances. This compound has strong binding interaction with VAL 734 and ASP 863 of the binding pocket along with hydrophobic interactions with LEU 726 and LEU 852. The hydroxyl group of ligand binds to the ASP 863 acting as H-donor making protein–ligand complex stable. In addition the delocalized pi electrons of the aromatic ring form unbreakable interaction with VAL 734 forming compound favorable inhibitor. The interaction detail is given in Table 3 and the binding interaction is shown in Fig. 7B.

The 1^st^ compound i.e. PH-1 has better interaction detail and may inhibit the activity of protein, the compound has a strong interactions with binding site residue MET 801 with several other interactions like with LEU 726, VAL734 and LEU 752. The ligand has carbonyl oxygen at position 6 satirically unhindered forming bonds with nitrogen of MET 801. The interaction detail, docking score is given in Table 2 whereas protein ligand interaction is shown in Fig. 7A.

### Molecular docking against VEGFR

**Fig. 8:**
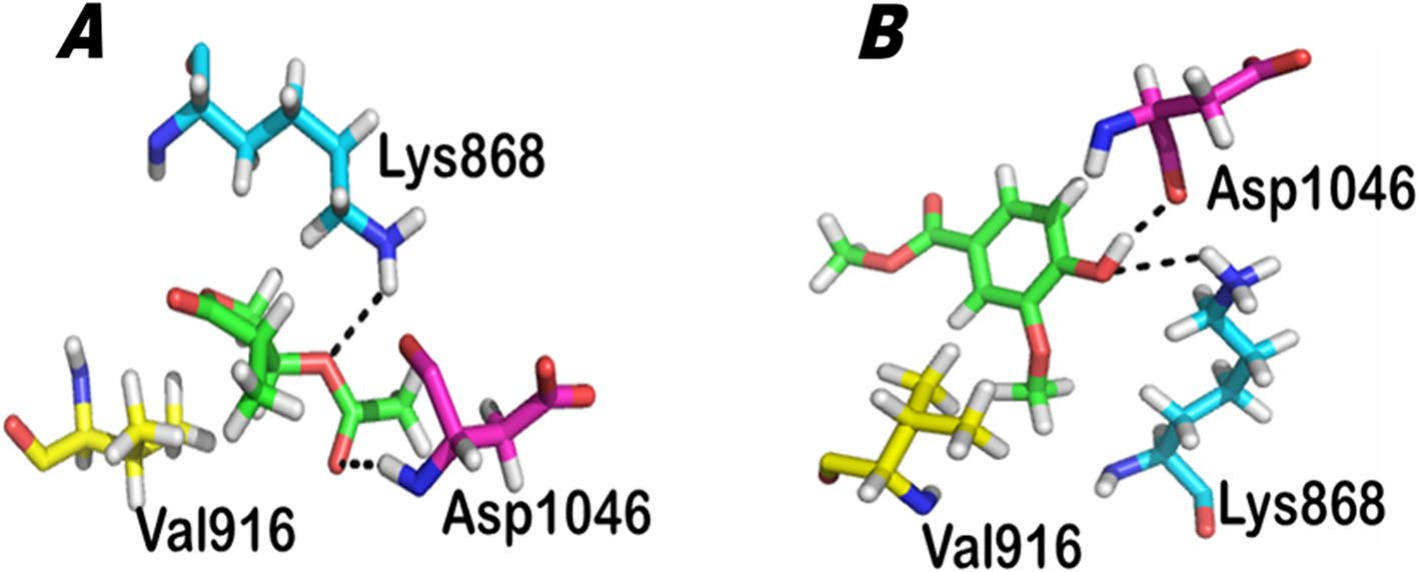
**A**, **B** Binding interactions of compounds with VEGFR. **A** Interaction with PH-1 and **B** interactions with PH-2. Protein ligand interaction diagram. The green color show ligand atoms

The comparative anti-angiogenic results of the PH-1 and PH-2 against the receptors VEGFR along with their respective docking scores are summarized in Table 4. As interaction detail given in the Table 4 explored that both the tested compounds have stronger interaction with VEGFR. Compound PH-1 has three strong intermolecular interactions with binding pocket residues Lys868, Val916 and Asp1046. The compound forms strong hydrogen bonding with Lys868 and Asp 1046 and a hydrophobic interaction with Val916. The compound has two electronegative carbonyl oxygen at carbon 9 and 10 which both formed hydrogen bond with HZ3 of Lys868 and with Nitrogen of Asp1046 forming a tight link with active site. The interaction diagram is shown in Fig. 8A. Interestingly the 2nd compound i.e. (PH-2) also formed three intermolecular interaction with active site residues Lys868, Val916 and Asp1046. The compound has a terminal OH group attached to aromatic ring form two hydrogen bond with oxygen of Asp1046 and HZ3 of Lys868. This hydroxyl group of the compound makes the compound to highly interact with active site residues may because the corresponding OH is in resonance with pi electrons of ring. Like 4-methyl-5-oxo-tetrahydrofuran-3-yl acetate it has also one hydrophobic interaction with Val916 making the complex more stable. The interaction detail is given in Table 4 and interaction geometry in Fig. 8B. The similarity in binding pattern of both the compounds may because both the compounds have similar electronegative oxygen atom/group which interacts in a much similar way with corresponding residues. Shortly from the above docking analysis one may expect that these compound may not have only the ability to bind to EGFR and HER2 but also equally to VEGFR and may act as anti-angiogenic inhibitor. So the molecular docking studies also support the in-silico anti-angiogenic mechanism of the isolated compounds.

**Table 4:** Protein ligand interaction detail of VEGFR with isolated compounds

**Samples**	**Docking Score**	**Interaction details**							
		Ligand	Number/position of atom	Receptor	Name of amino acid (residue)	Number of amino acid	Interaction	Distance (Å)	Energy (kcal/mol)
**PH-1**	-5.9865	O	10	N	ASP	1046	H-acceptor	3.11	-0.8
		O	9	HZ3	LYS	868	H-acceptor	2.7	-0.5
**PH-2**	-5.7853	O	7	O	ASP	1046	H-donor	2.72	-1.8
		O	7	HZ3	LYS	868	H-acceptor	2.6	-1.5

## Conclusions

We isolated two potent compounds which displayed substantial cytotoxicity against MCF-7, HeLA and NIH/3T3 cells. CAM assay revealed anti-angiogenic potentials and anti-tumor assay suggests tumor suppressing effect by our test samples. Molecular docking revealed the mode of action of compounds is mediated via inhibition of EGFR, HER2 and VERGR receptors. However, further detailed studies are required regarding the in-vivo efficacy of the tested compounds in these type of cancers.

## Supplementary Information

**Additional file 1.** Contains structures elucidation data of the compounds.

**Additional file 2.**

## Data Availability

The datasets used and/or analyzed during the current study are available from the corresponding author on reasonable request.
