# Supplementary material for: Cytotoxicity, anti-angiogenic, anti-tumor and molecular docking studies on phytochemicals isolated from Polygonum hydropiper L
Source: BMC Complement Med Ther. 2021 Sep 24;21:239. doi: 10.1186/s12906-021-03411-1 (PMC8464109; doi:10.1186/s12906-021-03411-1)
Supplement: Supplementary file 1 — Additional file 1. Contains structures elucidation data of the compounds. [file 12906_2021_3411_MOESM1_ESM.docx]

**Cytotoxicity, anti-angiogenic, anti-tumor and molecular docking studies on Phytochemicals isolated from *Polygonum hydropiper* L.**

Mater H. Mahnashi^1^*, Yahya S. Alqahtani^1^, Bandar A. Alyami^1^, Ali O. Alqarni^1^, Farhat Ullah^2^ , Abdul Wadood^3^, Abdul Sadiq^2^, Azam Shareef^3^, Muhammad Ayaz*^2^

**Mater H. Mahnashi^1^*** **Email:** matermaha@gmail.com

**Yahya S. Alqahtani^1^** **Email:** yahyasalqahtani0@gmail.com

**Bandar A. Alyami^1^** **Email:** alyamibandar1@gmail.com

**Ali O. Alqarni^1^** **Email:** aoqarni@gmail.com

**Farhat Ullah^2^** **Email:** [farhataziz80@hotmail.com](mailto:farhataziz80@hotmail.com)

**Abdul Wadood^3^** **Email:** [awadood@awkum.edu.pk](mailto:sadiquom@yahoo.com)

**Abdul Sadiq^2^ Email:** [sadiquom@yahoo.com](mailto:sadiquom@yahoo.com)

**Azam Shareef^3^ Email:**azamshareef46@gmail.com

**Muhammad Ayaz*^2^**  **Email:** [ayazuop@gmail.com](mailto:ayazuop@gmail.com)

^1^Department of pharmaceutical chemistry, college of pharmacy, Najran University, Najran, Kingdom of Saudi Arabia

^2^Department of Pharmacy, Faculty of Biological Sciences, University of Malakand, Chakdara, 18000 Dir (L), KP, Pakistan.

^3^Department of Biochemistry, Abdul Wali khan University Mardan

***Correspondence;***

**Dr. Muhammad Ayaz**, Department of Pharmacy, University of Malakand Khyber Pakhtunkhwa 18800, Pakistan.

**Tel:** +92-346-8004990,

**Email:**Ayazuop@gmail.com,

**ORCID:** https://orcid.org/0000-0002-4299-2445

**Professor Dr. Mater H. Mahnashi**,

Department of pharmaceutical chemistry, college of pharmacy, Najran University, Najran, Kingdom of Saudi Arabia

**PH-1: 4-methyl-5-oxo-tetrahydrofuran-3-yl acetate (MA-3)**

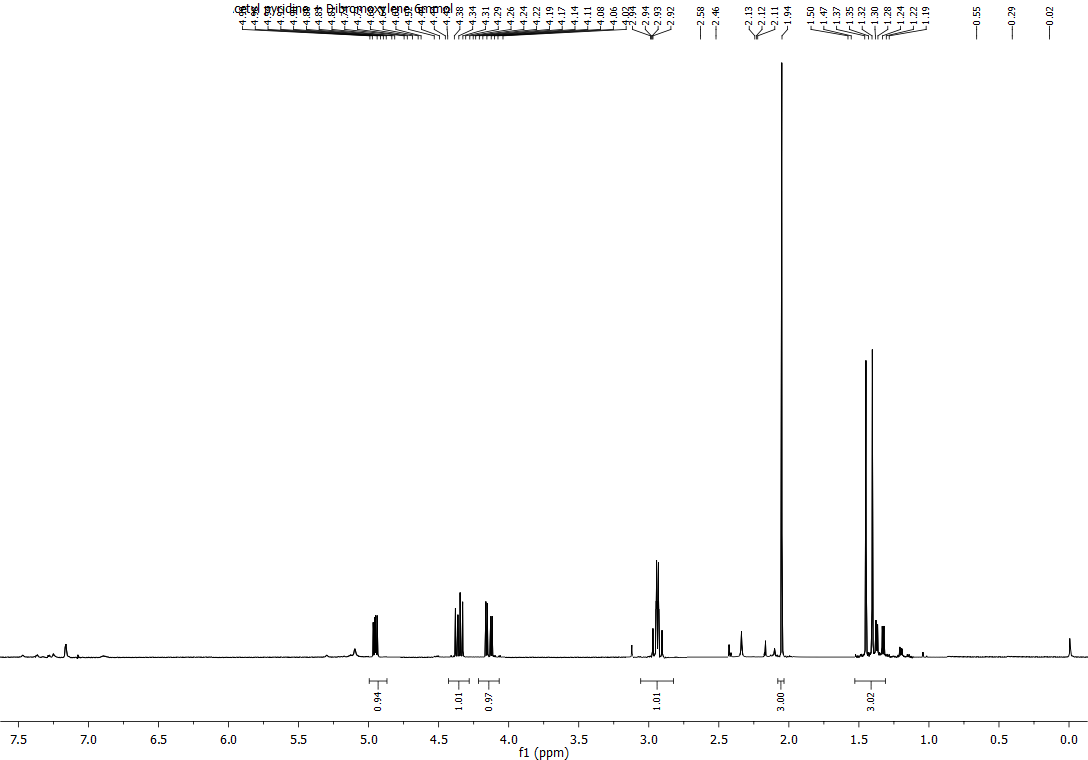


**Figure S1:**^1^H NMR of PH-1

**
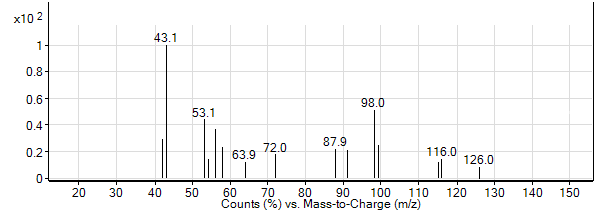
**

**Figure S2:** MS of PH-1

**Compound PH-2: Methyl 4-hydroxy-3-methoxybenzoate (MA-4)**

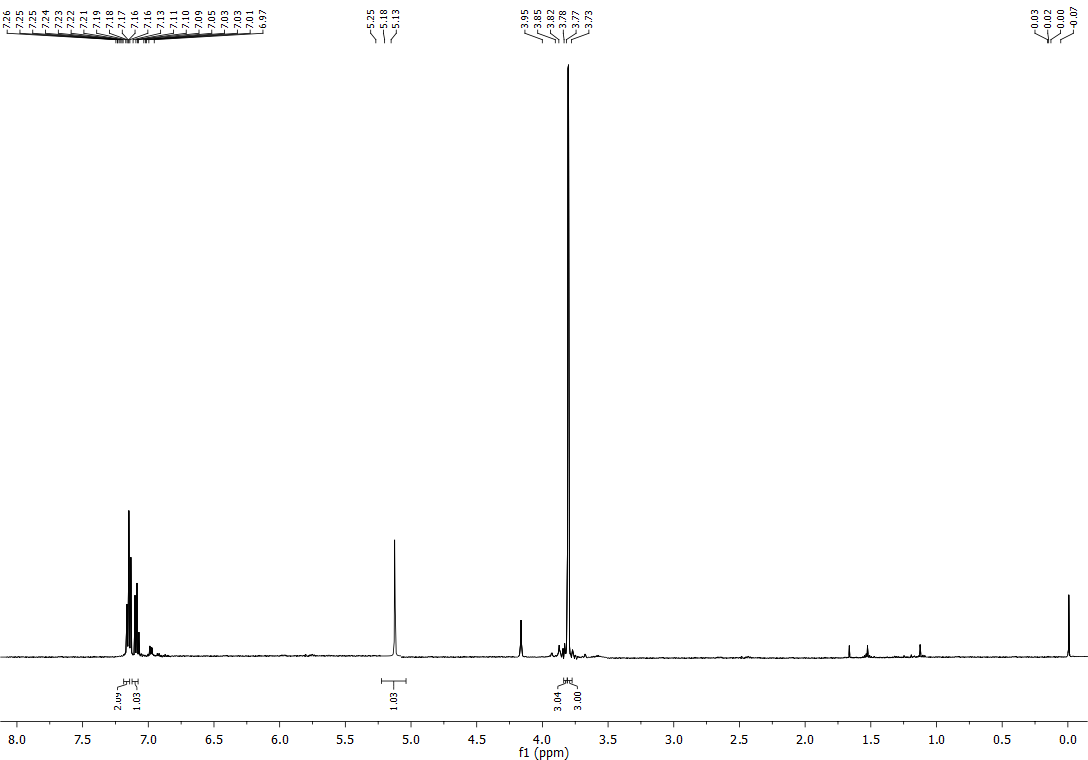


**Figure S3:**^1^H NMR of PH-2


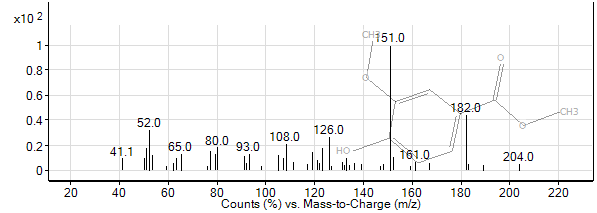


**Figure S4:** MS of PH-2
