# Supplementary material for: Cytotoxicity, anti-angiogenic, anti-tumor and molecular docking studies on phytochemicals isolated from Polygonum hydropiper L
Source: BMC Complement Med Ther. 2021 Sep 24;21:239. doi: 10.1186/s12906-021-03411-1 (PMC8464109; doi:10.1186/s12906-021-03411-1)
Supplement: Supplementary file 2 — Additional file 2. [file 12906_2021_3411_MOESM2_ESM.pdf]

Government of Pakistan

MINISTRY OF FOOD, AGRICULTURE AND CO-OPERATIVES

DEPARTMENT OF PLANT PROTECTION

PAKISTAN PLANT QUARANTINE ACT, 1976 AND PLANT QUARANTINE RULES, 1967 (AS ENFORCED UNDER P.Q. ACT, 1976) PLANT QUARANTINE)

PREFACE

The "Destructive Insects and Pests Act, 1914" was enacted by t former British Indian Government for preventing the introduction and spread of exotic pests and diseases which could be destructive to field crops, horticulture floriculture and forests. In exercise of the powers conferred by sub-section (1) Section 3, 4A and 4D of the said act, rules were framed by the then Central Government of undivided India. After Independence the act was adapted by the Government of Pakistan under Governor - General Order No. 4 in March, 1949. The Plant Quarantine Rules were slightly amended for Pakistan and corrected up to 15th September, 1962.

In order to make these rules fully effective under Pakistan conditions, it was felt necessary to revise the rules and update them as far as possible so that they are in conformity with the recommendations of the FAO International Plant Protection Convention, 1951 and rules and regulation of other countries. The Plant Quarantine Rules were revised and consolidated in 1966 under the provisions of the Destructive Insects and Pests Act and published in Government Gazette of Pakistan Extraordinary *vide* **SRO 129 (K) / 67**, dated 2nd January, 1967. With the rapid development of fast means of transport, increased trade relations and establishment of new air, land and sea routes, the movement of plants and plant material has increased manifold. The adapted Destructive Insects ands Pests Act, 1914, needed revision and modifications in the light of present advances in the field of Plant Protection and Plant Quarantine. The new Plant Quarantine bill entitled "Pakistan Protection Quarantine Act, 1976" was, therefore, enacted to safeguard the national crop wealth from destructive pests and diseases which are not know to occur in Pakistan.

The revised Plant Quarantine Rules now being printed as second impression are deemed to have been framed in exercise of powers conferred under Section 3, sub-sections (1), (2), (3) and Section 5 and Section 10 of the Pakistan Plant Quarantine Act, 1976, which is currently in force.

PAKISTAN PLANT QUARANTINE ACT, 1976

Whereas it is expedient to give effect in Pakistan to the International Plant Protection Convention, 1951, and to provide for matters connected therewith.

It is hereby enacted as follows:

1. **Short title, and commencement:**

(1) This Act may be called the Pakistan Plant Quarantine Act, 1976.

(2) It extends to the whole of Pakistan.

(3) It shall come into force at once.

2. **Definitions:**

In this Act, unless there is anything repugnant in the subject or context:

(a) "convention" means the International Plant Protection Convention, 1951;
(b) "crop" includes all agricultural or horticultural crops and all trees, bushes, grass or plants;
(c) "import" means the bringing or taking by sea, land or air across and customs frontier as defined by the Federal Government and includes transportation by any means from a one Province to another or from one place to another within a Province;
(d) "infection" means infection by any insect, fungus, or other pest injurious to a crop or plant;
(e) "pest" means any living stage of any or all insects, nematodes, slugs, snails, protozoa, or other invertebrate animals, fungi, bacteria, or other parasitic plants or reproductive parts thereof, virus or any organism, or any infections substance which may directly or indirectly injure or cause disease to any cop or plant; and
(f) "plant" means all species of plants or parts thereof whether living or dead including stems, branches, tubers, bulbs, corns, stock, bud-wood, cuttings, layers, slips, suckers, roots, green scum on stagnant pools, leaves, flowers, fruits and seeds.

3. **Power to prohibit or regulate import of certain articles:**

(1) The Federal Government may by notification in the official Gazette, prohibit, restrict or otherwise regulate, subject to such conditions as it may think fit to impose, import of any article or class of articles likely to cause infection to any crop or plant, or of any pest generally or any class of pests.

(2) A notification under this may specify any article or class of articles or any pest or class of pests either generally or in particular manner, whether with reference to the country of origin, or the route by which imported other otherwise.

(3) Any condition imposed under a notification under this section may require:
(a) compliance with the provisions of the Convention;
(b) examination by authorized persons of the goods, articles of plant origin and pests brought in or to be taken out;

(c) obtaining of certificate of non-infection ; and
(d) treatment or detention, confiscation and destruction of infected goods or articles of plant origin.

(4) **Operation of notification under section 3:**
A notification under section 3 shall operate as if it has issued under section 16 of the Customs Act, 1969 (IV of 1969), and the officers of customs shall have the same powers in respect of any goods, articles of plant origin or pests in respect of which such a notification has been issued as they have for the time being in respect of any article the importation of which is prohibited or restricted under the said Act, and all the provisions of that Act, shall have effect accordingly.

(5) **Goods and articles import or transportation of which prohibited not to be carried:**
When a notification has been issued under section 3, then not with standing anything contained in any other law for the time being in force, the person responsible for the booking of goods or parcels at any port, railway station, airport or other place:

(i) where the notification prohibits import, shall refuse to receive for carriage at, or to forward, or knowingly allow to be carried by sea, air, land, rail or other carrier form that port, station airport or place anything of which the import has been prohibited; and
(ii) where the notification imposes any condition upon, import, shall so refuse unless the prescribed documents showing that the conditions have been fulfilled are produced.

(6) **Penalty:**
Whoever contravenes or attempts to contravene any of the provisions of this Act, or of any rules made or notification issued thereunder, shall, without prejudice to any confiscation or penalty to which he may be liable under the provisions of the Customs Act, 1969 (IV of 1969), as applied by Section 4, be punished with fine which may extend to five hundred rupees and, upon any subsequent conviction, with imprisonment for a term which may to six months, or with fine which may extend to two thousand rupees, or with both.

(7) **Cognizance of offence:**
No court shall take cognizance of any offence under this Act except upon complaint in writing made by any officer authorized by the Federal Government in this behalf.

(8) **Indemnity:**
No suit, prosecution or other legal proceeding shall lie against any person for anything which is in good faith done or intended to be done under this Act.

(9) **Delegation of powers:**
The Federal Government may delegate to a Provincial Government, or to any officers or authority such Government, any of its powers under this Act insofar as it relates to the prohibition or restriction of bringing or taking or

taking into or out of any place form or to another within a Province or to the sale of goods or articles of plants origin imported in contravention of any of this provisions of this Act.

(10) **Power to make rules:**

(1) The Federal Government, may by notification in the official Gazette, make rules for carrying out the purposes of this Act.

(2) In particular and without prejudice to the generality of the foregoing power, such rules may provide for all or any of the following matters, namely:

(a) the method and manner of examination and treatment of goods and articles of plant origin likely to cause infection to any crop or plant;
(b) the form of certificate of non-infection;
(c) the documents to be produced before goods or articles of plant origin are accepted for carriage; and
(d) the fees to be levied under this Act.

(11) **Repeal:**

The Destructive Insects and Pests Act, 1914 (II of 1914), is hereby repealed.

GOVERNMENT OF PAKISTAN

MINISTRY OF FOOD, AGRICULTURE AND CO-OPERATIVES

DEPARTMENT OF PLANT PROTECTION

(Plant Quarantine Division)

PAKISTAN PLANT QUARANTINE RULES, 1967

(PLANT QUARANTINE)

MINISTRY OF FOOD, AGRICULTURE AND CO-OPERATIVES

(Agriculture Division)

Islamabad, the 2nd January, 1967

In exercise of the powers conferred section 3, sub-section (i) (ii) and (iii) and sections 4 and 5 of the Plant Quarantine Act, 1976 and in super session of rules, orders and notifications in this behalf, the Federal Government is pleased to make the following rules, namely:

1. (1) These rules may be called the Pakistan Plant Quarantine Rules, 1967.

(2) They shall come into force at once.

2. **Definitions:**
In these rules, unless there is anything repugnant in the subject or context.

(a) "act" means the Pakistan Plant Quarantine Act, 1976.

(b) "american cotton" all cotton produced in any part of the Western hemisphere (North, South and Central America and adjoining islands);

(c) "bale" means any pressed package of cotton of whatever size or density;

(d) "cotton" includes ginned cotton and droppings, strippings, fly and other waste products of a cotton mill other than yarn waste, but does not include cotton seed or unginned cotton;

(e) "department" means the Department of Plant Protection under Ministry of Food, Agriculture and Co-operatives (Agriculture Division);

(f) "director" means the Director of the Department;

(g) "disease" means any pathological abnormal condition of a plant caused by insects, mites, nematodes, protozoa, fungi, bacteria and viruses, recognizable by the presence of symptoms or of the organism inciting it;

(h) "disinfestation or disinfection" means any scientific treatment applied for the purpose of destroying or reducing any infection or infestation that may occur on, in or amongst plant materials;

(i) "exporter" includes any person who, whether as owner, consignor, agent to broker, is in possession of, or in any way entitled to, the custody or control of the plant;

(j) "form" means a Form annexed to these rule;

(k) "importer" means includes any person who, whether as owner, consignee, agent or broker, is in possession of, or in any way entitled to, the custody or control of the plant;

(l) "official certificate" means a certificate of plant health granted by the appropriate officer or authority in the country or origin;

(m) "plant pest" means any living animal or plant in any stage of its development known, suspected or liable to be harmful to the existence or growth of economic plants or to plant materials, whether by direct infestation or attach or by causing or spreading diseases in economic plants and known to infest land or waster, thereby preventing or obstructing its possible agricultural uses;

(n) "plant or plant material" means all species of plants or parts thereof, whether living or dead, including stems, branches, tubers, bulbs, corns, stocks, budwood, cuttings, layers, slips, suckers, roots, leaves, flowers, fruits, seeds, seedling and any other product of plant origin which has not been processed, such as bark, husk and peelings;

(o) "Plant Quarantine Officer" means any person authorized in writing by the Director to act on his behalf;

(p) "Plant Quarantine Division" means the Plant Quarantine Division of the Department;

(q) "prescribed port or point of entry" means the authorized routes, namely, Karachi Harbor, Karachi Airport, Lahore Airport, Lahore Railway Station, Waga (Lahore) border Jamrud (Peshawar) Land Border, Peshawar Airport, Chaman (Quetta) Land border, Quetta Airport or any other authorized land, sea air routes as may be declared by the Director;

(r) "entomologist (Quarantine)" means the Entomologist (Plant Quarantine) of the Department;

(s) "round bale" means a bale not exceeding 270 lbs. in weight; and

(t) "square bale" means a bale exceeding 270 lbs. in weight.

(2) All provisions referring to plant or plant materials shall apply also to all packing material used in packing or wrapping such plant or plant material.

IMPORT

3. Import of Plant material:

No person shall import any plant or plant material which may be a source be a source or medium of infestation or infection by diseases and pests destructive to agriculture or medium for the introduction of noxious weeds, except under a valid import permit obtained prior to such importation in Form I issued by the Director or the Entomologist (Quarantine) and except through the ports or points of entry.

4. Plant material for which special permit is required:

Not withstanding anything contained in rule 3, plant material likely to carry new complex of pests or diseases may be imported into Pakistan in limited quantities by special permit in Form I for the purpose of introducing new varieties and propagating stock from countries which maintain regular plant quarantine and inspection service.

Provided that the importation of all plant material falling under this category shall be made only through Karachi Harbour, Karachi Airport, and shall be subject to any restriction imposed on the permit to import the same.

5. Application for permit to import plant material:

1) Before any plant or plant material is imported, an application for permit shall be submitted to the Director or to the Entomologist (Quarantine).
2) All such applications shall be signed by the person who intends to import the pant or plant material or his duly authorized agent and shall specify:

(a) the kind and quantity of plant or plant material;
(b) the country and locality of origin;
(c) destination;
(d) the name and address of the consignor and the consignee;
(e) means of transport;
(f) the prescribed port or point of entry;
(g) the prescribed for which the plant or plant material is proposed to be imported e.g., consumption propagation or processing.

6. Notice of arrival by the importer:

The importer shall inform the Director or the Plant Quarantine Officer, of the probable date of arrival of the plant or plant material at the prescribed port or point of entry and shall, on arrival of the plant or plant material, notify to the Director the number of the permit, name of ship or vessel, date of arrival, country of origin and locality where grown, and the character and quality of the plant or plant material.

7. Refusal and revocation of permits:

A permit to import plant or plant material may b revoked if, in the opinion of the Director or the Entomologist (Quarantine), the importer has willfully contravened any provision of these rules or there is reason to believe that the plant or plant material will be imported in violation of the provision of these rules.

8. Foreign Certificate of Inspection:

(1) A plant or plant material the shipment of which originates from a country maintaining a plant quarantine service shall be accompanied by an official certificate.

(2) In the case of countries which do not maintain a plant quarantine service, the certificate of inspection of the plant or plant material must be accompanied by a declaration of ht exporter or shipper concerned to the effect that the plant or plant material does not originate from a place where injurious insects or plant diseases were prevalent and has not been kept or stored in places infested with injurious insects or infested by diseases and plant pests, and that all treatment, fumigation, disinfestation required prior to shipment has been done under technical supervision.

(3) Persons who import any plant or plant material shall submit the official certificate of the declaration of the exporter or shipper to the effect mentioned in sub-rule (2) to the Plant Quarantine Officer for his perusal and records but this will, however, not preclude inspection by the Plant Quarantine Officer, if deemed necessary.

(4) The shipper or consignee shall submit an affidavit to the Plant Quarantine Officer to the effect that the required official certificate or declaration mentioned in sub-rule (2) will be presented within 30 days from the receipt of the shipment.

(5) Shipments arriving without a valid official certificate or declaration mentioned in sub-rule (2) and without permit shall either be confiscated and destroyed after making an order in Form II or returned to the port of origin at the expense of the importer.

(6) Shipments arriving at any prescribed port or point of entry accompanied with an official certificate or declaration but without import permit or vice versa may be released at the Director's discretion after post-entry examination and issuance of an import permit.

(7) Small consignments of plant or plant material brought as passenger's accompanied or unaccompanied baggage with or without official certificate or declaration and import permit may be released only if they conform to the requirements of these rules and after examination by the Plant Quarantine Officer and issuance of an import permit.

9. Freedom of plant material from San, Soil or Earth:

An imported plant or plant material shall be free from sand, soil, saw dust or earth and the plant roots, rhizomes and tubers shall be washed thoroughly when possible re-packed in such sand, soil earth as is certified by the duly authorized officer of the exporting country to have been sterilized and rendered safe.

10. Packing material:

All packing material employed in the importation of nursery stock and other plants, including any material of plant origin used for packing purposes, shall be examined and approved by the Plant Quarantine Officer as to their safety for such use.

11. Plant material imported by post:

Plant or plant material imported through the post shall be inspected by the Plant Quarantine Officer upon notification or their presence at the Post Office. The plant or plant material shall be handled in the same manner as those coming through authorized routes and may be released or confiscated after inspection and treatment through the Postal authorities.

12. Importation of insects and other animals, pests , plant diseases and cultures for scientific and allied purpose:

(1) No person shall import from any country any harmful, living insects, animals, birds, fungi, fungal cultures, obnoxious weed plants or their propagating material except in accordance with the following provisions, namely:

Any living stage of the numerous small invertebrate animals elongated invertebrates lacking appendages, commonly referred to as worms, for example, nematodes, any form of protozoa; any form of fungi such as rusts, smuts and moulds, any form of bacteria; any form of viruses, or any form of similar or allied organisms which may directly or indirectly affect, injure or cause disease in plants, unless:

(a) the proposed importation is to be used for scientific, educational, commercial or industrial purposes only;

(b) an application is submitted to the Director stating the names and addresses of the consignor and consignee, the scientific name of pests or diseases, the institution or place of origin, quantity, number of containers, the purpose by whom or the institution where the material will be used;

(c) the importation is only to the routed through Karachi harbour, and Karachi Airport.

(d) the forwarding label issued with the permit designating the route through which the importation is permitted has been forwarded by the importer to the shipper and attached to the outside of each consignments; and

(e) every importer authorized under this sub-rule is subject to inspection at the prescribed port or point of entry and may be held for further examination and refused entry or destroyed if, in the opinion of the Plant Quarantine Officer, such importation is found to include insects or other organisms not specifically covered by the permit.

(2) No person shall import exotic beneficial insects, birds, animals both vertebrate and invertebrate (Mollusca, Crustacea Myriopoda, Insecta) for scientific, educational, industrial or medicinal purpose except under, and in accordance with the terms of or an authority from the Director.

(3) No person shall import useful exotic plant species including useful micro-organisms and their propagating materials unless he has obtained the prior permission of the Director and the articles imported are covered by an official certificate.

13. Jurisdiction Authority of Quarantine Officer:

(1) Were on inspection, any imported plant or plant material is found to be infested or infected with any plant pests or diseases the plant or plant material and containers thereof shall be destroyed in the presence of the Plant Quarantine Officer or returned to the shipper after treatment, at the discretion of the Director, and where any carrier is found to be infested with ant pests or is suspected to be so infested it shall be treated to the extent and in the manner deemed necessary by the Plant Quarantine Officer. When any action is necessary due notice shall be given to the owner or his agent and any risks associated with such treatment shall be the responsibility of the owner.

(2) No person shall obstruct an Officer of the Department in the due discharge of his duties; and no person shall refuse to permit the making of any examination required under these rules or refuse to carry out the instructions of an Officer relative to the effective control of any insect, pests and diseases.

IMPORT RESTRICTIONS OR PROHIBITIONS

14. Potato:

(1) Potatoes shall not be imported into Pakistan by any means from any country, where the three serious pests, namely, Black wart (**Synchytrium endobioticum**), Golden Nematode (**Globodera rostochiensis**), Colorado potato beetle (**Leptinotarsa decemlineata**) have either been prevalent or reported to have occurred unless they are accompanied by official certificate from the country of origin declaring that the crop from which the consignment is derived was not grown in the vicinity of unhealthy potato crops and was inspected by a duly authorized official Phytopathological Service of the country of origin and found free from all injurious insect, pests and diseases mentioned above was recorded within 2 kilometers radius of the place where the potato crop was grown during the past twelve months.

(2) In order to guard against the importation of the pests and diseases mentioned in sub-rule (1), the Director shall take the following precautions, namely:

(a) as far as possible, import only of seed potato resistant to wart disease shall be permitted;
(b) the importation of seed potatoes shall be permitted from those countries where the aforesaid pests and diseases are not present;
(c) import only seed potatoes certified by the Phytopathological Service of the exporting county to have been produced in areas within the country free from the pests and diseases shall be permitted;
(d) seed consignments should be free from shoots, leaves, roots and soil sticking to tubers;

(e) import of potato seeds should be restricted only to certified seed free from virus diseases except virus X; and
(f) import of shoots, leaves and green parts of potato shall not be permitted.

15. Rubber:

1) Any plant of the Hevea shall not be imported into Pakistan, unless:

(a) the importation is made for scientific purposes;
(b) written permission has been granted for each consignment of plants by the Department and the importation is in accordance with such special conditions as may be imposed by the Director or the Entomologist (Quarantine) in granting such permission;
(c) the plant has been disinfected and freed of any original soil in the country of origin and is free from pests and diseases, and consignment of plants is accompanied or covered by an official certificate specifying clearly that the above requirements has been fulfilled; and
(d) each consignment is addressed to the Director or any person authorized by him.

2) The importation into Pakistan of any plant of the genus Hevea capable of further growth or propagation (excluding seed) is prohibited from the American tropics or from any other country in which South American leaf blight (**Dothidella ulei**) is present, unless, in addition to the requirements of sub-rule (1), such plant has been grown for an adequate period at a plant quarantine station for Hevea at a place approved by the Director and situated outside the American tropics and any other country in which South American leaf blight (**Dothidella ulei**) is present, and each consignment of such plats is accompanied or covered by an official certificate to the effect that the above requirements have been fulfilled, and signed by the Officer-in –Charge for such quarantine station.

3) The importation into Pakistan of any seed of any plant of the genus Hevea is prohibited from the American tropics or from any other country in which South American leaf blight (**Dothidella ulei**) is present unless, in addition to the requirements of sub-rule (1), such seed, having been examined and again disinfected at a place approved by the Director and situated outside the American tropic and any other country I which South American leaf blight (**Dothidella ulei**) is present, has been repacked with new packing materials in new containers and unless each consignment of such seed is accompanied or covered by an official certificate to the effect that the above requirements have been fulfilled and signed by the Officer-in- charge of these operations.

4) The importation into Pakistan of any plant or plant material of the genus Hevea not capable of further growth or propagation (such as fresh or dried herbarium specimens) is prohibited, unless, in addition to the requirements of clauses (a), (b) and (d) of sub-rule, (1), the Director is satisfied that such plant or plant material is required for a legitimate special purpose and that such plant or plant material has been sterilized in the country of origin by a method satisfactory to the Director.

5) The importation into Pakistan of any plant or plant material other than the genus Hevea, capable of further growth or propagation and originating in the American tropic or in any other country in which South American leaf blight (**Dothidella ulei**) is present, is prohibited unless written permission has been granted for each consignment of such plant or plant material by the Director and the importation is in accordance with such special conditions as may be imposed by the Director in granting such permission.

6) The Department shall ensure that any plat of the genus Hevea imported through it for further growth or propagation is grown under control for such periods as will ensure that it is free from all pests and diseases before it is released.

7) All budwood and other propagation material of Hevea rubber shall be dusted with sulphur before shipment and the budwood shall be dipped in a Mercuric Chloride Solution for a brief period immediately before use to eliminate **Cidium hevea**, and immediately after dipping the budwood shall be washed throughly in running water. (A solution suitable for this purpose consists of 0.2 per cent mercuric chloride in 50 per cent methyl (or ethyl) (alcohol).

8) All the imported propagating material not used for budding and the packing material imported with it shall be destroyed by burning.

16. Sugarcane:

Importation of sugarcane is prohibited provided that the Director may authorize the importation of small quantities of plant or plant material, subject to the following conditions:

i) that the importation is made for scientific purposes;

ii) that the imported planting material is grown at a recognized post entry quarantine station for an adequate period before release for large-scale planting; and

iii) that the usual official certificate accompanying the consignment should bear an additional declaration to the effect that the planting material was inspected in the field and was found to be free from injurious pests and diseases and particularly from the following diseases, namely, Sugarcane Mosaic virus, Sugarcane Fiji disease virus, Pine apple disease (**Ceratocystis paradoxa**) Sereh disease, gummosis, (**Xanthomonos vasculorum**), ratoon virus and grassy shoot virus.

17. Tobacco:

Unmanufactured tobacco, either raw or cured, shall not be imported into Pakistan unless, in addition to the usual official certificate required, it is accompanied by an additional declaration that it is free from **Ephestia elutilla** or that the pest does not exit in the country of origin. The import of tobacco seedlings is prohibited seed should be accompanied with the usual official certificate which must also declare that

the seeds have been treated by immersion for 15 minutes in 0.1% solution of silver nitrate.

18. Citrus Plants:

Citrus plant and cutting shall not be imported into Pakistan unless, in addition to the general requirements under rules 3 to 6, the following conditions are fulfilled:

i) the importation is made for scientific purpose;
ii) the imported plants or cuttings are grown at a recognized post entry quarantine station for a period adequate to ensure their freedom from virus diseases; and
iii) the usual official certificate bears an additional declaration to the effect that the plants are free from Malsecco caused by **Deuterophoma tracheiphila** and that the disease does not exist in the country in which they are grown.

19. Coffee Plants, seeds and beans:

coffee plants, coffee seeds and coffee beans shall not be imported into Pakistan except with the special permission of the Director, who shall take all measures necessary to ensure that such coffee plants, seeds or beans as are permitted by him are free from plant diseases and injurious insect pests.

Provided that this prohibition shall not apply to roasted and ground coffee.

20. Banana:

Importation of banana suckers and fruits prohibited from South West of India and any other country where the diseases have been reported to have occurred to prevent the spread of Bunchy Top virus disease and Panama disease due to the fungus (**Fusarium oxysoporum var.**)

21. Coconut:

Importation of coconut plant or plant material into Pakistan is prohibited from Caribbean area, Jamaica, Haiti, Florida, Ghana, Togoland, Philippines, British Guiana, West Indies, South India, Guam and any other areas where the following diseases of obscure origin are known to occur:

(a) Red Ring (Aphelenchoides cocophilus);
(b) Lethal Yellowing;
(c) Kaincope disease;
(d) Cadang cadang;
(e) Bronze leaf Wilt;
(f) Root (Wilt) disease;
(g) Guam coconut disease.

But its from other countries can be made subject to the following conditions:

i) importation of coconut materials shall under permit be made only in small quantities, restricted to unsprouted nuts from which the perianth has been removed.

ii) the seed nuts shall be accompanied by an official certificate from an

appropriate authority in the country of origin that the seeds come from trees showing no signs of diseases'

iii) upon arrival, the seeds shall be fumigated or treated by any other method considered appropriate by the Director to remove the risk of introducing insects; and

iv) the importation seed nuts shall be planted in individual containers in isolated quarantine for a period of one year. Any diseased seedlings, together with containers and planting media shall be burnt.

22. Groundnut:

Importation into Pakistan of groundnut seedling from U.S.A., U.S.S.R., West Indies, and China is prohibited to prevent the introduction of groundnut (Puccinia arachidis), (Sphaceloma arachidis) and other posts. Decorticated seeds may, however, be imported only under permit and usual official certificate.

23. Maize:

Importation into Pakistan of seedlings from Canada, U.S.S.R., U.S.A., Mexico, Porto Rico and Italy is prohibited but the seeds may be imported under permit and if the consignment is accompanied with official certificate stating that the crop was not infected by (Xanthomonas stewart).

24. Tea:

Importation into Pakistan of the tea vegetative material from as area where the disease caused by **Exobasidium reticulatum** is know to occur (Japan) and from any county where phloem necrosis virus exists (Ceylon) is prohibited. Tea seed can be imported only under permit and shall be accompanied by an official certificate as well as by a certificate of field inspection. In addition, these shall be treated, before their shipment and after arrival, with an appropriate fungicide and insecticide.

25. Allium spp.

Plants and bulbs of onion, garlic, shallot, leek and chive shall not be imported into Pakistan unless these are certified as free from smut disease (Urocystis caepulae).

26. Cocoa and Theobroma spp. Cocoa and other Theobroma spp. (including seed in the unmanufactured state) may be imported for research and propagation by Government controlled institutions only from countries other than Africa, the West Indies and Ceylon. The consignment shall be accompanied by a consignor's certificate stating fully the origin of the plants. Official certificate shall certify that they were inspected and found free of Pod rot (Monilia rorei) mealy pod (Treachysphaera fructigena) and witches broom (Marasmius perniciosus) and that swollen shoot and other virus diseases do not occur in the country of origin. Plant are subject to inspection and fumigation at the prescribed port or point of entry.

27. Soil and Rooted Plants:

The imported into Pakistan of soil or any other unsterilized rooting media such as compost, humus or forest litter, capable of carrying pathogens is prohibited.

28. Cotton:

(1) Unginned cotton shall not be imported into Pakistan.

(2) Cotton seed shall not be imported save for experiment purposes by officer in Pakistan authorized in this behalf by the Central Government at the ports of Karachi and shall not be imported in quantities exceeding one pound in weight in any one consignment and shall be fumigated at the port of entry with an appropriate fumigant;

Provided that if the cotton seed is accompanied by a certificate from the Government Entomologist of the country of origin to the effect that the seed and its container have been treated in such a way as to destroy all insect life, the seed shall be examined on importation by Plant Quarantine Officer and shall not be required to be re-fumigated unless such examination shows that re-fumigation is necessary.

(3) American cotton shall not be imported into Pakistan by any means except with the special permission of the Director and shall not be so imported save through the port of Karachi and subject to the following conditions:

(a) on or before the departure of a ship carrying a consignment of American cotton for Karachi from the port which the cotton is consigned, the consignee or shipper shall ascertain the name of the ship, the probable date of its arrival in Karachi and number of square and round bales of American cotton contained in the consignment and shall furnish this information to the Director not less than fourteen days before the arrival of the ship at Karachi.

Provided that where the American cotton is loaded for Karachi at Port Said or at a European port the ordinary length of voyage from which is less than three weeks, it shall be sufficient to furnish the information not less than ten days before the arrival of the ship at Karachi.

(b) on arrival of the ship carrying consignment of American cotton at Karachi, the cotton shall be disinfected in such manner and by such authority as the Director may direct: and thereafter the release order in Form III shall be issued;

(c) no vessel shall discharge American cotton during a period of rain, mist or drizzle;

(d) no vessel carrying American cotton shall enter the territorial water of Pakistan without the previous permission in writing of the Director in Form IV for which application shall be made to the Director at least fourteen days before the expected arrival of the vessel;

(e) no American cotton or any other cotton, the fumigation of which is required by these rules or any other cotton which may have been in contact therewith or in proximity thereto, shall be landed without fumigation, except at a special landing place provided by the Trustees

of the Port of Karachi and approved by the Director for the reception of unfumigated American cotton.

(4) Cotton other than American cotton, which is imported into Pakistan in a vessel carrying American cotton and is loaded in the same batch as any bale or bales of American cotton shall be deemed to have been I contact therewith or in proximity thereto and shall be subject to the restrictions and conditions specified in these rules.

(5) Samples of American cotton imported by parcel post or as ships parcel, not exceeding 20 lbs, each in weight, shall be fumigated at the Custom House on arrival by an approved fumigated of the Department.

(6) Transit movement or transhipment of American cotton or American cotton samples is permitted if shipments are accompanied by official certificates and are so packed that insects cannot enter or escape.

(7) All cotton the fumigation of which is required by these rules shall be at the sole risk of the importer during landing transhipment and fumigation; and no liability for any loss or damage due to fumigation shall attach to the Government or its agents.

EXPORTS

29. Inspection and Certificate for exports:

All persons who intend to export plant materials must submit to the Department, an application for inspection of plant or plant material before the dispatch of such consignments. All such plant or plant material meant for export shall be inspected for any injurious insects and plant diseases, and plants and plant materials which are found to be free from injurious insects and diseases shall be certified in V and it found to exporter or destroyed at the option of the exporter. In either case, the cost shall be borne by the exporter.

30. Application for Inspection of Plant Material for export:

All persons who intend to export plant or plant material shall subject to the Director or Entomologist (Quarantine), an application for inspection of the plant or plant material they desire to export within a reasonable time. The application should be made at least a day before the shipment in the case of non-perishable goods, so as to allow proper inspection and certification.

31. Certification of Plant material for export:

(1) (a) If the plant or plant material upon inspection are found to be free from plant diseases and injurious insects, a certificate in Form V shall be issued by the Director or Entomologist (Quarantine) to the exporter to accompany the shipment.

(b) If the Director, or as the case may be, the Entomologist (Quarantine) considers necessary he may, before issuing a certificate under clause (a), require the disinfestation or disinfection of plants or plant materials by fumigation

through a person, firm, agency organization or company registered with the Department.

(c) The Department may register any persons, firm, agency organization or company for carrying out disinfestation or disinfection of plant or plant materials on such terms conditions and on payment of such fees as it may determine (as per annexure I).

(d) No official certificate shall be granted for plant or plant material which have been from or mixed with other plants which are diseased or infested.

(e) The official certificate shall not be granted for any plant or plant material intended for shipment to a country in which their entrance is absolutely prohibited.

(2) All risk or damages or any kind associated with or resulting from fumigation or other treatment shall be at the risk of the owner.

(3) The certificate implies that the plant or other matter was inspected by a duly authorized officer of the Department and was fund to conform to the inspection standards or procedures associated with the issuance of official certificates tags or other documents.

32. The list of officers authorized to inspect and grant the official certificate is at Form VI.

33. Plant Material in Transit:

(1) The provisions of these rules shall also apply to the plant and plant material under transit through Pakistan by land, air or sea or their transhipment at the discretion of the Director.

(2) The shipments and consignments must, however, be accompanied by official certificate, issued by the proper official or the authority from the country of origin and shall be so packed that there are no chances of any insect pests and disease escaping from the packages or the containers.

MINISTRY OF FOOD AND AGRICULTURE

(Agriculture Wing)

Islamabad, the 17th September, 1973

S.R.O. 1343 (I)/73. – In exercise of the powers conferred by sub-section (1) of section 3, sections 4A and 4D of the Destructive Insects and Pests Acts, 1914 (II of 1914), the Federal Government is pleased t direct that the following amendment shall be made in the Destructive Insects and Pests Rules, 1966 (Plant Quarantine), namely:

In the aforesaid Rules, in rule 31, in sub-rule (1), after clause (a), the following new clauses (aa) and (aaa) shall be inserted, namely:

"(aa) If the Director, or as the case may be, the Quarantine Entomologist considers necessary he may, before issuing a certificate under clause (a), require the disinfestation or disinfection of plant or plant materials by fumigation through a person, firm, agency, organization or company registered with the Department.

(aaa) The Department may register any person, firm, agency, organization or company for carrying out disinfestation or disinfection of plant or plant material on such terms and conditions and on payment of such fees as it may determine."

EXTRACT FROM THE GAZETTE OF PAKISTAN, EXTRA.,
PART II, SEPTEMBER 20, 1973

Annexure I

TERMS AND CONDITIONS FOR REGISTRATION OF COMMERCIAL FUMIGATORS

The following terms and conditions are laid down by the Department for the registration of Commercial Fumigators in connection with fumigation of plants and plant material meant for export. The Department may from time to time amend or modify the terms and conditions through notification in the Government required:

Registration required:

1. No commercial fumigator shall engage in, perform or offer to perform any fumigation unless he is registered with the Department.

2. The Department may fix the registration fee and prescribe any terms upon which registration may be granted, renewed, suspended or cancelled.

(a) Under the terms referred herein the Director will prescribe the procedure and conditions for fumigation and aeration of buildings, vehicles, carries etc.
(b) Fix the amount and type of insurance or surety that shall be carried or furnished by a Commercial fumigator against any expected loss or damage resulting from careless performance of fumigation.
(c) Prescribe the form and contents of the notice under section 7.
(d) Prescribe the medical facilities and first Aid requirements which shall be available at the site of fumigation to meet emergencies.
(e) Specify or prohibit the use of any fumigant.
(f) Regulate installation, operation and use of any machine, apparatus or equipment used for fumigation.
(g) Deal with any other matter necessary or advisable to carry out effectively the intent and purpose of this registration.

Responsibility of Fumigator:	Every Commercial Fumigator shall with respect to any fumigation be responsible, for the acts or omissions of his employers assistants or agents during the period of fumigation and aeration.
Liability Insurance:	4. A commercial fumigator shall insure against any liability accruing as a result of faulty or defective fumigation.
Deputation of Plant Quarantine Officers / officials:	5. The Director shall appoint or depute Plant Quarantine officials/ officers to supervise the fumigation at the specified time, date and place. These officials / officers will certify the effective fumigation before a phytosanitary certificate is issued by him.
Note of Fumigation:	6. The commercial fumigator shall deliver a notice in writing at least 24 hours before starting fumigation to a responsible person at any premises, building, vehicle or carrier.
Contents of notice:	7. Every notice state:<br>(a) the address where fumigation is to be carried out.<br>(b) that there is danger of poisonous gas.<br>(c) The date and time when it is proposed to commence fumigation.<br>(d) That the occupants are to vacate ad remain out of buildings, vehicles, carries, premises during the period of fumigation and aeration, and<br>(e) Such other information as may be prescribed by the department.
Duty of Fumigator:	8.The commercial fumigator shall ensure that the building or premises, vehicles or carriers under fumigation are unoccupied during the period of fumigation and aeration.
	9. Any officer of the Department of Plant Protection, Ministry of Food Agriculture may at any time enter any building, vehicle, carrier, premises where he has reason to believe that fumigation had been, is being or is about to be carried out.

Suspension of Registration: 10. On finding that the Commercial Fumigator is not complying with agreed terms and conditions his license is liable to be suspended for period of not more than six months.

PROCEDURAL DETAILS FOR REGISTRATION

(i) An application for registration shall be made to Central Government on prescribed from.

(ii) The Director on receipt of applications from the Central Government shall scrutinize and examine the application or his establishment on his/ their knowledge of fumigation, toxic qualities, antidotes, forms and methods of application of fumigant, of the identification, life history, characteristics and control of insects.

(iii) After examination a report shall be submitted to the Central Government with recommendations of the Director for or against registration.

(iv) Registration shall not be granted unless the Director is fully satisfied with regard to:

(a) Technical know-how of fumigation.
(b) Supplies and equipment, fumigants, Fumigation Chambers, Tarpaulins etc.
(c) Safety equipment, respirators, leak detectors etc.

(v) Each Registration shall be valid for a period of two years from the date of issue of registration letter from the Central Government.

(iv) Renewal of registration may be applied with in 30 days of its expiry.

(vii) Fumigation shall be done in a manner that leakages do not occur and the premises is not rendered unsafe for the surrounding areas. All such material, food, water etc. likely to absorb the gas shall be removed from the premises.

(viii) A placard showing “DANGER” shall be displayed at the premises where fumigation is in progress.

(ix) Fee to be charged for services rendered by the according to the schedule to be prescribed later on.

(x) Commercial fumigators shall submit a certificate of fumigation giving the name of chemical, dose, time of exposure and other such information to the Plant Quarantine Organization after the treatment is over and successfully completed.

(xi) *Registration Fee*: A nomial amount of Rs. 100.00 (Ruppees one hundred) shall be charged as a Registration Fee.

JEHANGIR SHAH JOGEZAI,
Joint Secretary.

THE GAZETTE OF PAKISTAN

No. 43: Karachi, Wednesday, October 28, 1987

Islamabad, the 9th September, 1987

ADDITIONAL CONDITION FOR THE IMPORT OF SUNFLOWERS SEED

No. F. 1-24/87-Pest: In exercise of the powers conferred by Section 3, subsections (1) and (3) a and c of the Plant Quarantine Act, 1976, the Federal Government is pleased to notify that the Sunflower seeds imported into the country should be free from borne diseases namely Downy Mildew (*Plasmopara halstedi*), Rust (*Puccinia helianthi*), Sclerotinia Head rot (*Sclerotinia sclerotiorium*), Septoria leaf spot (*Septoria helianthi*), Verticillium Wilt (*Verticillium Spp.*), as well as weed seeds and accompanied with Phytosanitary Certificate from the country of origin with an additional declaration that the plants and plant materials are free from the above named diseases.

Sd/-
SAEED AMED KHAN,
Section Officer,

FORM I

(See rules 3 & 4)

GOVERNMENT OF PAKISTAN

MINISTRY OF FOOD, AGRICULTURE AND CO-OPERATIVES

Department of Plant Protection

Wire: “LOCUSWAR”

Your Reference:

No.
Jinnah Avenue, Malir Halt,
Karachi-27, the

IMPORT PERMIT

SPECIAL IMPORT PERMIT

Permission is hereby granted to ______________________________

to import ______________________________

Grown in ______________________ through ______________________

The importation will be regulated by the following conditions:

(1) The consignment is accompanied with health certificate from the country of origin declaring the plant or plant material has been rendered free from injurious insects and pests by effective fumigation.

(2) On arrival of the consignment it will be examined and fumigated by the technical staff of this Department stationed at ______________________ and will only be released if they are found free from disease or plant pest otherwise the same will be destroyed.

(3) The permit is valid for the three months from the date of its issue.

(4) No plant shall be imported into Pakistan by means of letter or sample post.

DIRECTOR / ENTOMOLOGIST (QUARANTINE).

To, ______________________________

____

Copies to:

1. The collector of Customs, Customs House.
2. The Chief Inspector of Customs.
3. The Assistant Entomologist (Quarantine).

DIRECTOR / ENTOMOLOGIST (QUARANTINE).

FORM I

(See rules 3 & 4)

No. **Form No. DPP-PQ/I**

GOVERNMENT OF PAKISTAN

MINISTRY OF FOOD, AGRICULTURE AND CO-OPERATIVES

Department of Plant Protection

Book No. (Plant Quarantine Division) Serial No.

Ring:
Wire: 'LOCUSWAR'

Jinnah Avenue, Malir Halt,
Karachi-27, the

Your Reference: Your No. ………………………………… dated ………………………...

IMPORT PERMIT

In exercise of the powers conferred by the Destructive Insects and Pests Act, 1914 (as adapted for Pakistan). permission is hereby granted to:

Name & Address: ……………………………………………………………………………

to import …………………………………………………………………………………….

……………………………………………………………………………………………….

……………………………………………………………………………………………….

grown in …………………………………. by …………………………… through the port of

……………………………………. The importation and release of the consignment shall be subject to:

1. The consignment is free from soil and accompanies with a valid Phytosanitary Certificate, issued by a competent authority of the country of origin declaring it to be free from insect, pests and diseases or rendered so by effective treatment.
2. Quarantine examination and treatment at the port of entry after arrival or any other place designated by the Director of Plant Protection or any Officer so authorized by him and shall thereafter be released only after issuance of release order from competent Plant Quarantine Authority.
3. Payment of such examination and / or treatment charges and as may be levied under rules.
4. Additional conditions on (reverse).

Importation (s) made otherwise than in according with the conditions listed above shall be liable to confiscation and destruction.

This permit is valid till …………………………..

DIRECTOR / QUARANTINE ENTOMOLOGIST

To

1. The Permittee ………………………………………………………………..
   ………………………………………………………………..
2. The Asstt. Collector Customs …………………………………………………..
   …………………………………………………..
3. The Asstt. Quarantine Entomologist …………………………………………...
   …………………………………………..

FORM II

[See rules 8 & (5)]

GOVERNMENT OF PAKISTAN

MINISTRY OF FOOD, AGRICULTURE AND CO-OPERATIVES

Department of Plant Protection

(Plant Quarantine Division)

Phone : 480111-5 lines

Wire: “LOCUSWAR”

No.
Jinnah Avenue, Malir Halt,
Karachi-27, the

The plant or plant material mentioned below having been imported without the prior permission of the Director, Department of Plant Protection, and unaccompanied with a valid official certificate, as required by the Pakistan Plant Quarantine Rules, 1967, is hereby ordered to be confiscated and destroyed.

Particulars of the plant or plant material	Consignee
____________________	____________________

ENTOMOLOGIST (QUARANTINE).

The Assistant Collector of Customs,

Copy for information:

Form-No. DPP-POIII

GOVERNMENT OF PAKISTAN

MINISTRY OF FOOD, AGRICULTURE AND CO-OPERATIVES

Department of Plant Protection

(Plant Quarantine Division)

Wire: 'LOCUSWAR'

Ring.

No.
Plant Quarantine Station
Dated,

Your Reference:

Dated:

RELEASE ORDER (General)

Plants and plant products specified below were examined by a duly authorized officer of this Department and were found apparently free from injurious insects and diseases. The consignment may now be released to the consignee:

____

____

QUALIFYING REMARKS ……………………………………………………………………

DESCRIPTION OF ARTICLES …………………………………………………………………

Director

To,

____

____

Copy to:

FORM III

[See rules 28 (3) (b)]

GOVERNMENT OF PAKISTAN

MINISTRY OF FOOD, AGRICULTURE AND CO-OPERATIVES

Department of Plant Protection

Phone : 480111-5 lines, 226417

Wire: "LOCUSWAR"

No.

Jinnah Avenue, Malir Halt,

Karachi-27, the

RELEASE ORDER

This is to certify that a consignment of ___________bales of American cotton imported through s. s. ______________ was examined and fumigated by a duly authorized Officer of this Department. This is now apparently free from exotic insects and pests. The Consignment, is therefore, released.

QUALIFYING REMARKS:

Fumigation complete
on ________________

DIRECTOR / ENTOMOLOGIST (QUARANTINE).

To,

M/s ___________________________

____

____

Copies to :

(1) The Collector of Customs, House, Karachi.

(2) The Traffic Manager, Karachi Port Trust, Karachi.

(3) The Assistant Entomologist (Quarantine), Karachi, Seaport.

DIRECTOR / ENTOMOLOGIST (QUARANTINE).

FORM IV

[See rules 28 (3) (d)]

GOVERNMENT OF PAKISTAN

MINISTRY OF FOOD, AGRICULTURE AND CO-OPERATIVES

(Plant Quarantine Division)

Wire: "LOCUSWAR"
Fax : 9248673

No.
Jinnah Avenue, Malir Halt,
Karachi-27, the

Phone : 9248612-15(4 lines)
Your Reference:

Dated:

ANCHORAGE PERMIT

Permission is hereby granted to the s.s. ______________________________expected on ________________, carrying raw cotton to anchor at Karachi Harbour. Necessary examination / fumigation will be conducted by a duly authorized officer of this Department on its arrival subject to the condition that no vessel shall discharge raw cotton during a period of rain, mist or drizzle.

for Plant Protection Advisor
and Director General

To,

M/s. ___________________________

____

____

Copies to :

The Permitee

for Plant Protection Advisor
and Director General

Original
Form No. DPP-PQ/V

Government of Pakistan

MINISTRY OF FOOD, AGRICULTURE AND CO-OPERATIVES

Department Of Plant Protection

Karachi, Pakistan

PHYTOSANITARY / HEALTH CERTIFICATE

Book No. ………………… Serial No. ……………………

No. ……………………………

Date …………………………..

PAKISTAN PLANT QUARANTINE SERVICE

To ………………………………………………
(Plant Protection Organization)

Of ………………………………………………
(Importing Country)

DESCRIPTION OF CONSIGNMENT

Name and address of exporter ……………………………………………………………………………
………………………………………………………………………………………………………
Declared name and address of consignee …………………………………………………………………
………………………………………………………………………………………………………
Number and description of packages ……………………………………………………………………
………………………………………………………………………………………………………
Distinguishing marks …………………………………………………………………………………
………………………………………………………………………………………………………
Place of origin ………………………………………………………………………………………
Declared means of conveyance ………………………………………………………………………
Declared port of entry …………………………………………………………………………………
Name of produce and quantity declared …………………………………………………………………
………………………………………………………………………………………………………
………………………………………………………………………………………………………
Botanical name of plants ………………………………………………………………………………
………………………………………………………………………………………………………

This is to certify that the plants or plant products described above have been inspected and found free from quarantine pests and substantially free from other injurious pests; and that they are considered to conform with the phytosanitary regulations of the importing country.

DISINFESTATION AND / OR DISINFECTION TREATMENT.

Date ……………………… Treatment ……………………………………………………………………
Chemical (Active ingredient) ……………………………………………………………………………
Duration and temperature ………………………………………………………………………………
Concentration ………………………………………… Additional information ………………………
Date of final inspection ……………………………………

Additional Declaration:

Place of issue ……………………………………………
Name and designation ……………………………………
Of authorized Officer ……………………………………
……………………………………………………………
Date ………………………………………………………
……………………………………………………………
(Signature)

This certificate applies only to injurious insects and plant diseases of the growing crop which are readily capable of detection at the time of shipment. No. liability shall attach to the Department of Plant Protection, to any officer or representative of that Department with respect to this certificate. Erasures, cutting, overwriting or any change in the text are not allowed.

FORM VI

(See rules 32)

GOVERNMENT OF PAKISTAN

MINISTRY OF FOOD, AGRICULTURE AND CO-OPERATIVES

Department of Plant Protection

List of Officers authorized to inspect

And grant the official certificates

1. Director, Department of Plant Protection, Karachi.
2. Deputy Director (Quarantine), Department of Plant Protection, Karachi.
3. Entomologist (Quarantine), Department of Plant Protection, Karachi.
4. Assistant Entomologist (Quarantine), Department of Plant Protection, Karachi.
5. Mycologist, Assistant Mycologist (Quarantine), Department of Plant Protection.

Signed:

PCPPK-S/546/94-95/Met. Deptt.-14-12-94-200 Books
